# In Vitro Experimental and Computational Studies of the Vasorelaxant Effects of Quinazolin-4(3*H*)-one and Its Interaction with α1-Adrenergic Receptors

**DOI:** 10.3390/cimb48090963

**Published:** 2026-09-20

**Authors:** Yassine Rhazi, Ismail Bouadid, Fatimazahra Guerguer, Mohammed El Mesky, Houda Lamssane, Asmae Nakkabi, Mohamed Bakhouch, Malak Yahia Qattan, Noorah A. Alkubaisi, Samir Chtita, Mourad A. M. Aboul-Soud, John P. Giesy, Mohamed El Yazidi, Mohamed Eddouks

**Affiliations:** 1 Engineering Laboratory of Organometallic, Molecular and Environment, Faculty of Sciences Dhar EL Mahraz, Sidi Mohamed Ben Abdellah University, Atlas, P.O. Box 1796, Fez 30000, Morocco; 2 Team of Ethnopharmacology and Pharmacognosy, Faculty of Sciences and Techniques Errachidia, Moulay Ismail University of Meknes, Boutalamine, P.O. Box 509, Errachidia 52000, Morocco; 3 Laboratory of Analytical and Molecular Chemistry, Faculty of Sciences Ben M’Sik, Hassan II University of Casablanca, P.O. Box 7955, Casablanca 20360, Moroccosamirchtita@gmail.com (S.C.); 4 Laboratory of Materials Engineering for the Environment and Natural Resources, Faculty of Sciences and Techniques, University of Moulay Ismail of Meknès, Boutalamine, P.O. Box 509, Errachidia 52000, Morocco; m.elmesky@edu.umi.ac.ma (M.E.M.);; 5 Laboratory of Applied Organic Chemistry, Faculty of Science and Techniques, Sidi Mohamed Ben Abdellah University, Fez 30050, Morocco; 6 Laboratory of Bioorganic Chemistry, Department of Chemistry, Faculty of Sciences, Chouaïb Doukkali University, P.O. Box 24, El Jadida 24000, Morocco; bakhouch.m@ucd.ac.ma; 7 Department of Health Sciences, College of Applied Studies, King Saud University, P.O. Box 4545, Riyadh 11451, Saudi Arabia; 8 Department of Botany and Microbiology, College of Science, King Saud University, Riyadh 11451, Saudi Arabia; 9 Center of Excellence in Biotechnology Research, College of Applied Medical Sciences, King Saud University, Riyadh 11433, Saudi Arabia; 10 Department of Veterinary Biomedical Sciences and Toxicology Centre, Western College of Veterinary Medicine, University of Saskatchewan, Saskatoon, SK S7N 5B4, Canada; 11 Department of Integrative Biology and Center for Integrative Toxicology, Michigan State University, East Lansing, MI 48824, USA; 12 Department of Environmental Sciences, Baylor University, Waco, TX 76706, USA

**Keywords:** hypertension, blood pressure, cardiovascular, pharmaceutical, drug, α1-adrenergic receptor antagonist, molecular docking, DFT-MEP-ADME-Tox

## Abstract

The problem of hypertension continues to be a major health challenge in the world, and the need to develop new and safer vasorelaxant agents continues to be a necessity. A compound with known pharmacological benefits that is yet to be examined for its role in blood pressure regulation, quinazolin-4(3*H*)-one, was synthesized and characterized, and then its vasorelaxant effects were investigated. In vitro vasorelaxation effects were evaluated on isolated rat aortic rings that had been pre-contracted with Epinephrine (EP) and Potassium chloride (KCl). An integrated in silico approach was employed to elucidate the compound’s interactions and properties. The AutoDock Vina (Version 1.5.7) tool was used for molecular docking. Following rigorous protein and ligand preparation, key binding modes at the active sites of the alpha-1 adrenergic receptor (α1-AR) and L-type calcium channels were identified. Quantum chemical properties were calculated by Gaussian 09 using Density Functional Theory (DFT). A combination of the B3LYP functional and the 6-311G (d,p) basis set was used. Molecular Electrostatic Potential (MEP) analysis was performed using GaussView. ADME-Tox, bioavailability, and pharmacokinetic properties were predicted using SwissADME and pkCSM. Quinazolin-4(3*H*)-one produced concentration-dependent relaxation of EP-pre-contracted aortic rings, regardless of the presence of the endothelium. Moreover, the compound significantly and concentration-dependently inhibited sustained EP-induced contractions. These findings indicate an inhibitory effect on EP-induced vascular contraction, but do not establish a specific α1-adrenergic receptor-mediated mechanism. Molecular docking analysis suggested a favorable potential interaction of quinazolin-4(3*H*)-one with α1-AR, although this computational prediction does not confirm a functional receptor-mediated effect. DFT and MEP analyses indicated favorable electronic properties and charge distribution that may contribute to molecular interactions. In silico ADME-Tox evaluation predicted favorable drug-like properties, including oral bioavailability and good absorption, with no major toxicity alerts. Overall, quinazolin-4(3*H*)-one represents a promising lead compound for further investigation of its vasorelaxant and potential antihypertensive properties. Further pharmacological and in vivo studies are required to elucidate its precise mechanism of action and establish its safety and therapeutic potential.

## 1. Introduction

Despite the many advances that have been made in the field of medicine, the danger of cardiovascular disease is as pertinent as it has ever been and is a serious concern in the world of global health [1]. High blood pressure (HBP) or hypertension is a prevalent disease that affects global public health, which is known as the ‘silent killer’ because it often appears with few symptoms. Due to the damage it does to the body’s organs when not treated, hypertension has a high mortality. The clinical manifestations include ventricular hypertrophy, congestive heart failure, and the acceleration of atherosclerosis, cerebrovascular, renal, and retinal problems [2]. Mechanically, blood pressure is determined by the interplay between how much blood the heart pumps (cardiac output) and the resistance encountered by that blood as it moves through the body. It is this resistance that is determined by the tone of the arteries, which is primarily governed by signals from the sympathetic nervous system that lead to their contraction. This makes it clear that stabilizing blood pressure within normal ranges is the most effective way to protect the long-term health of the heart and blood vessels [3]. Clinicians can use various medications to control blood pressure, including older diuretics and newer drugs, such as ACE inhibitors, beta blockers and calcium channel blockers. If these conventional treatments do not work, specialized second-line drugs such as clonidine or minoxidil can be added to the pharmaceutical therapies, allowing personalized strategies to be developed based on the individual requirements of patients. However, the clinical application of these molecules reveals a complex reality. Drugs have the two-faced, “Janus effect” of having undesirable, adverse side effects in addition to the desired pharmacological responses. Although their ability to lower blood pressure is undeniable, they often come with severe or at least uncomfortable side effects or contraindications. Because they often compromise patients’ long-term adherence to treatment, these iatrogenic limitations pose a significant challenge [4].

The pathophysiological mechanisms that contribute to the development of hypertension include increased vascular resistance, which is largely dependent on decreased vascular diameters due to increased vascular contraction and remodeling [5]. The arterial system plays a crucial role in regulating blood pressure by influencing vascular resistance. There are three major factors that affect blood flow resistance. These factors include the diameter of the blood vessel, the length of the blood vessel, and the viscosity of blood. Of all these factors, the diameter is the most important because it is subject to rapid changes due to contraction and relaxation [5,6]. As a result, the relaxation of smooth muscle in the blood vessels is one of the strategies used in the management of hypertension. Therefore, it was considered essential to pursue and develop novel vasorelaxant agents that exhibit minimal side effects.

**Figure 1:**
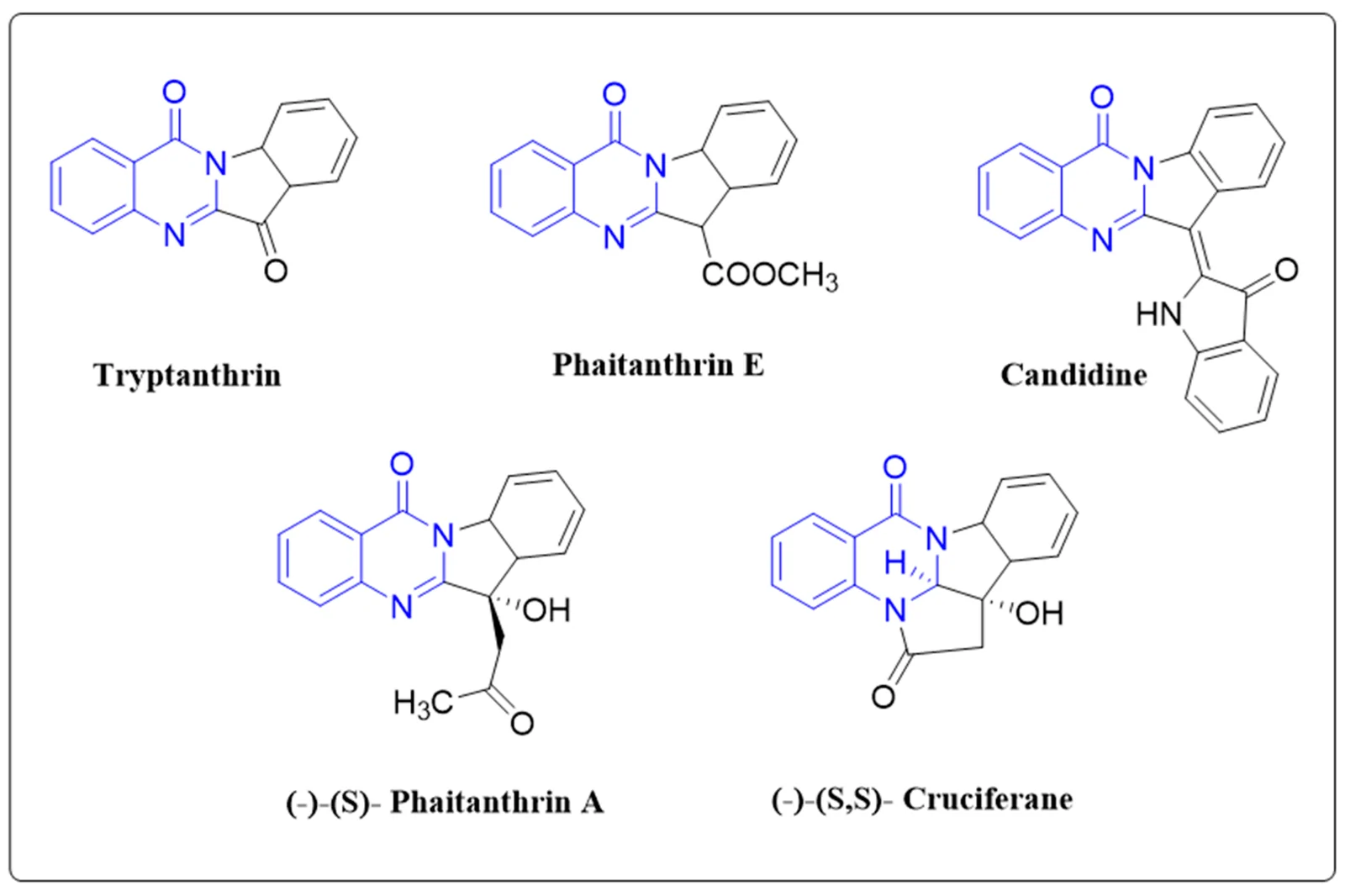
Natural products incorporating the quinazolinone core structure. The quinazolinone core is highlighted in blue to indicate its presence in the natural products.

Quinazolin-4(3*H*)-ones are nitrogen-containing heterocycles (Figure 1) that form the core structure of various natural products [7,8]. Numerous compounds in this class have demonstrated various biological activities, including vasorelaxant [9,10], antimicrobial, anticancer, analgesic, anti-inflammatory, anticonvulsant, antitubercular, antioxidant, and antileishmanial effects [11,12]. It is worth noting that the first-generation α1-adrenergic receptor antagonists in clinical use, including prazosin, terazosin, and doxazosin, are all quinazoline derivatives built on the 4-amino-6,7-dimethoxyquinazoline core, with high-affinity α1 binding in this series generally dependent on a basic piperazine or amide side chain attached at the 4-position [13]. Quinazolin-4(3*H*)-one, the compound investigated here, is structurally distinct from this classical pharmacophore: it lacks the 4-amino substituent, the 6,7-dimethoxy groups, and the piperazine/amide side chain considered essential for nanomolar α1 affinity in the prazosin-type series. The present study therefore examines the unsubstituted parent quinazolinone scaffold itself, rather than a functionalized quinazoline bearing the canonical basic side chain, in order to isolate the intrinsic contribution of the core ring system to vasorelaxant activity and α1-AR interaction.

In this study, quinazolin-4(3*H*)-one was synthesized by use of a previously published protocol [14,15]. The purified material was then evaluated for its vasorelaxant properties and elucidating its mechanisms of action. Additionally, molecular docking analysis, quantum chemical investigations, and in silico drug-likeness and ADME-Tox evaluations were conducted to assess the potential of quinazolin-4(3*H*)-one as a therapeutic candidate for arterial hypertension treatment.

## 2. Materials and Methods

### 2.1. Chemistry

Characterization of Quinazolin-4(3*H*)-one: yield: 92%; m.p.: 214–216 °C; IR (ν, cm^−1^): 3204 (N-H), 1705 (C=O); ^1^HNMR (300 MHz, DMSO- d6, δ in ppm): 12.26 (s, 1H, NH); 8.12 (s, 1H, CH); 8.15 (d, 1H, HAr, J = 6 Hz); 7.81 (dd, 1H, HAr, J = 5.7 Hz); 7.67 (d, 1H, HAr, J = 7.5 Hz); 7.52 (dd, 1H, HAr, J = 6 Hz). ^13^CNMR (75 MHz, DMSO-d6, δ in ppm): 161.23 (C=O); 145.9 (N=CH); 149.16; 134.8; 127.6; 127.2; 126.3; 123.08. ESI-QTOF-MS (*m*/*z*): accurate mass calculated for [C_8_H_7_N_2_O+H]^+^ = 147.0558 while the mass measured was 147.0555.

### 2.2. Animals

Healthy adult male albino rats of the Wistar strain weighing between 150 and 250 g were used for the experiment. These rats were supplied by the experimental center of Missour, Morocco. The rats were placed in separate plastic cages and were maintained in normal conditions. The rats were supplied with a normal pellet diet commonly used in laboratories on an ad libitum basis. The rats were given a minimum of three weeks to better acclimate to stress.

### 2.3. Chemical Reagents and Drugs

Details regarding chemicals and reagents are provided in the Supplementary Materials.

### 2.4. Vascular Relaxation: Measurement and Mechanistic Study

The Supplementary Materials contains the whole methodology and specific experimental techniques.

### 2.5. Statistical Analysis

Data are expressed as mean ± SEM. Statistical analyses were performed using two-way analysis of variance (ANOVA), followed by Bonferroni’s multiple-comparison test, as appropriate. A value of *p* < 0.05 was considered statistically significant. The inhibitor experiments were exploratory in nature, and no formal a priori power calculation or equivalence test was performed. Therefore, non-significant differences were not interpreted as evidence of equivalence or as definitive evidence of the absence of an effect. Rather, they indicate that none of the tested inhibitors produced a statistically detectable attenuation of quinazolin-4(3*H*)-one-induced vasorelaxation under the experimental conditions used. The possibility that smaller effects were not detected because of limited statistical power cannot be excluded.

### 2.6. Molecular Docking

To better understand the in vitro study, a molecular docking study was carried out to evaluate the binding affinities and modes of interaction of quinazolin-4(3*H*)-one with two major targets for its vasorelaxant effects: the alpha-1 adrenergic receptor (PDB ID: 7YMH) and L-type calcium channels (PDB ID: 8HMB) [16,17]. The protein structure was obtained from the RCSB Protein Data Bank and was refined using the Swiss PDB Viewer (Swiss-PdbViewer) [18], which involves the removal of hetero atoms, removal of water molecules, addition of missing hydrogens, and other appropriate modifications. The reference compounds and quinazolin-4(3*H*)-one were modeled using ChemOffice software, which was then utilized in Avogadro for geometric optimization using the MMFF94 force field [19]. They were subsequently incorporated into AutoDock Vina [20], a popular and well-validated utility for molecular recognition simulations. To further improve the accuracy of the results, the parameters of the calculation grid were also focused on the active sites of target proteins (Table 1). After the simulations were finished, BIOVIA Discovery Studio was used to display and analyze the ligand-protein interactions [19].

### 2.7. Quantum Chemical Investigation

Quantum chemical properties of quinazolin-4(3*H*)-one and reference drugs were computed by utilizing the Gaussian 09 program and Density Functional Theory (DFT) method with the hybrid function B3LYP and 6-311G (d,p) basis set. Using the HOMO-LUMO energies of the compounds, the parameters calculated were: ΔE (ELUMO − EHOMO), ionization potential (IP = −HOMO), electron affinity (EA = −LUMO), electronegativity (χ = [IP + EA]/2), chemical hardness (η = [IP − EA]/2), chemical softness (S = 1/η), chemical potential (μ = −[IP + EA]/2), and electrophilicity index (ω = μ^2^/2η). Additionally, theoretical results for the molecular electrostatic potential (MEP) of the compounds were obtained. The GaussView (Version 6.0) software was used to visualize the molecular orbitals and electrostatic potential surfaces [20].

### 2.8. Drug-likeness and ADME-Tox Evaluation

In addition to information on their intended pharmacological effects, for efficient drug discovery, it is essential to conduct an exhaustive evaluation of the optimal pharmacokinetic properties and toxicology of drugs in line with the “drug-likeness” criteria [21,22]. In silico screening methods for absorption, distribution, metabolism, excretion, and toxicity (ADME-Tox) have a significant impact on the reduction in the likelihood of late-stage failures and the refinement of the selection process for the most promising candidates [23]. Therefore, the new quinazolin-4(3*H*)-one molecule was systematically screened to determine its drug-likeness properties along with oral bioavailability and pharmacokinetic profile. This in silico characterization was carried out using the SwissADME and pkCSM reference platforms, thus allowing for rigorous predictive analysis regarding its potential biological behavior [24,25].

## 3. Results

### 3.1. Synthesis and Characterization of Quinazolin-4(3H)-one

**Scheme 1:**
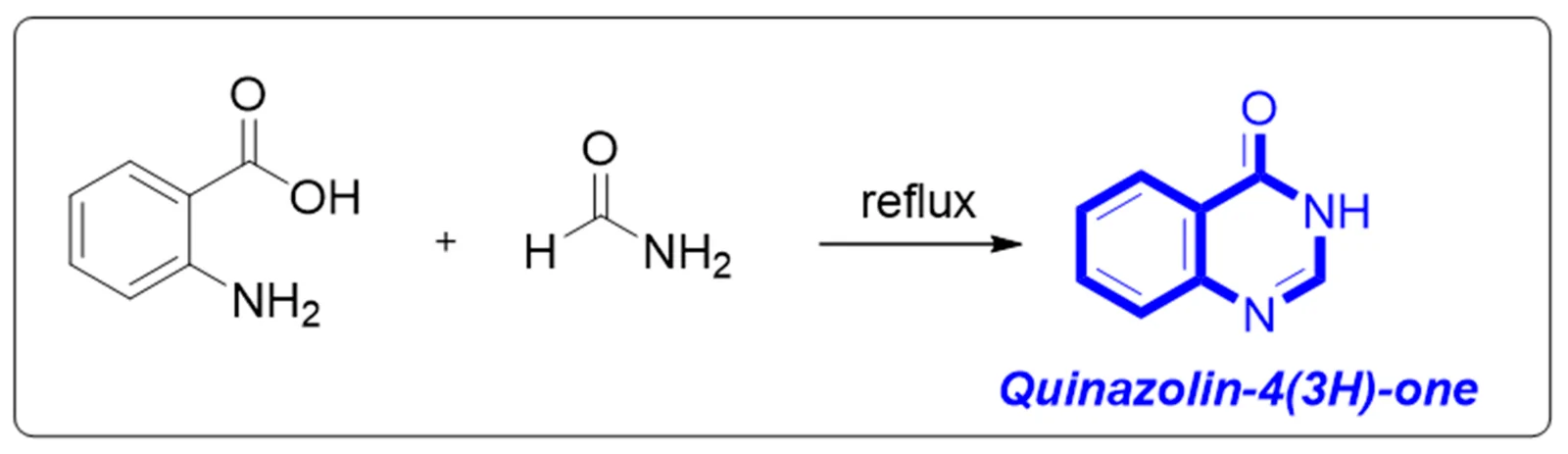
Methodology for synthesizing quinazolin-4(3*H*)-one scaffolding.

The quinazolin-4(3*H*)-one core was synthesized by the condensation reaction of anthranilic acid and formamide. The reaction mixture was refluxed for 6 h in good yields of the expected product (Scheme 1).

The structure of the compound was verified using several spectroscopic methods. The IR spectrum in Figure S1 revealed two key characteristics: a band at 3204 cm^−1^ that is linked to the vibration of the N-H bond and a prominent band at 1705 cm^−1^ that is indicative of the carbonyl group (C=O) of the amide function integrated into the ring. ^1^H NMR (Figure S2) analysis showed characteristic signals including a singlet at 12.26 ppm (NH), a singlet at 8.12 ppm (methine proton of the pyrimidinone ring), as well as four signals between 7.52 and 8.15 ppm (aromatic protons). The ^13^C NMR spectrum in Figure S3 allowed for the identification of the amide carbon (C=O) at 161.23 ppm, the methine carbon (CH) at 145.9 ppm, and the aromatic carbons between 123.08 and 149.16 ppm. Mass spectrometry confirmed the structure with an accurate molecular mass [M + H]+ of *m*/*z* 147.0555, in accordance with the molecular formula C_8_H_7_N_2_O (Figure S4).

### 3.2. Vasorelaxant Activity

#### 3.2.1. Quinazolinone-4(3*H*)-one-Induces Endothelium-Independent-Relaxation in Isolated Aortic Rings Rat

**Figure 2:**
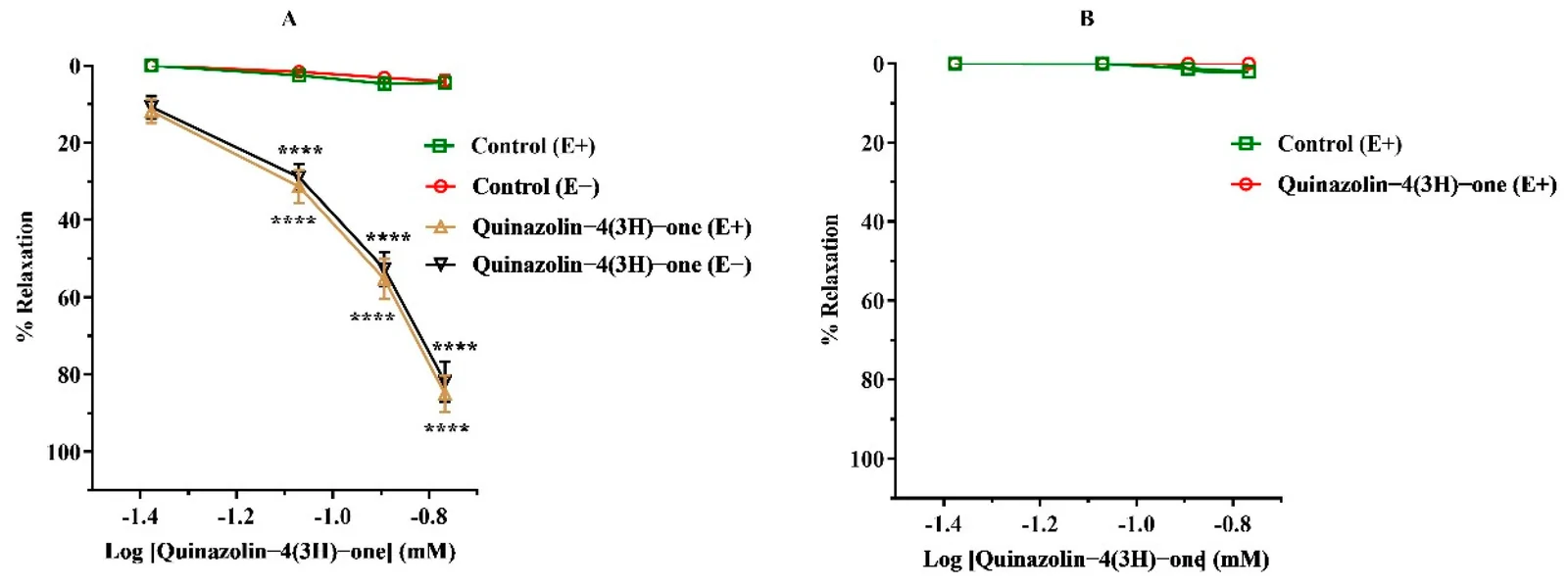
The concentration-response relaxation Impact of quinazolin-4(3*H*)-one (0.042, 0.085, 0.128 and 0.171 mM) on aortic rings pre-contracted by epinephrine (EP, 10 µM) (**A**) with (E+) or without (E_) vascular endothelium (panel **A**). The figure displays the vasorelaxation response in endothelium-intact aortic rings pre-contracted with KCl (80 mM) (panel **B**). Control groups received the vehicle alone (distilled water), added cumulatively in volumes equivalent to those used for quinazolin-4(3*H*)-one. Data reflect mean ± SEM (*n* = 6). **** *p* < 0.0001 vs. control.

Quinazolin-4(3*H*)-one-induced vasorelaxation was evaluated in a series of in vitro studies of rat aortic rings, analyzing relaxation responses to four concentrations (0.042, 0.085, 0.128, and 0.171 mM) in a dose-dependent manner (Figure S5). Experiments were conducted on pre-contracted aortic rings using either EP (10 µM) or KCl (80 mM) as the contracting agent (Figure 2). As illustrated in Figure 2A, quinazolin-4(3*H*)-one significantly relaxed both endothelium-intact and endothelium-denuded aortic rings pre-contracted by EP, with a significant effect starting at the second dose (0.085 mM, *p* < 0.0001). Two-way ANOVA revealed significant effects of concentration [F(3, 44) = 170.9, *p* < 0.0001, partial η^2^ = 0.921], experimental group [F(3, 44) = 392.3, *p* < 0.0001, partial η^2^ = 0.964], and their interaction [F(9, 44) = 47.48, *p* < 0.0001, partial η^2^ = 0.907]. Bonferroni’s multiple-comparisons test confirmed significant differences between quinazolin-4(3*H*)-one treated and corresponding control rings at the effective concentrations (adjusted *p* < 0.0001). The maximal relaxation (Rmax) was comparable between the endothelium-intact and endothelium-denuded groups, measuring 84.98 ± 4.56% and 81.87 ± 5.23%, respectively, with no statistically significant difference observed. In contrast, as shown in Figure 2B, quinazolin-4(3*H*)-one did not induce significant vasorelaxation in endothelium-intact aortic rings pre-contracted by KCl (80 mM).

#### 3.2.2. Effect of Different Inhibitors in Quinazolin-4(3*H*)-one-Induced Rat Aortic Rings Vasorelaxation

**Figure 3:**
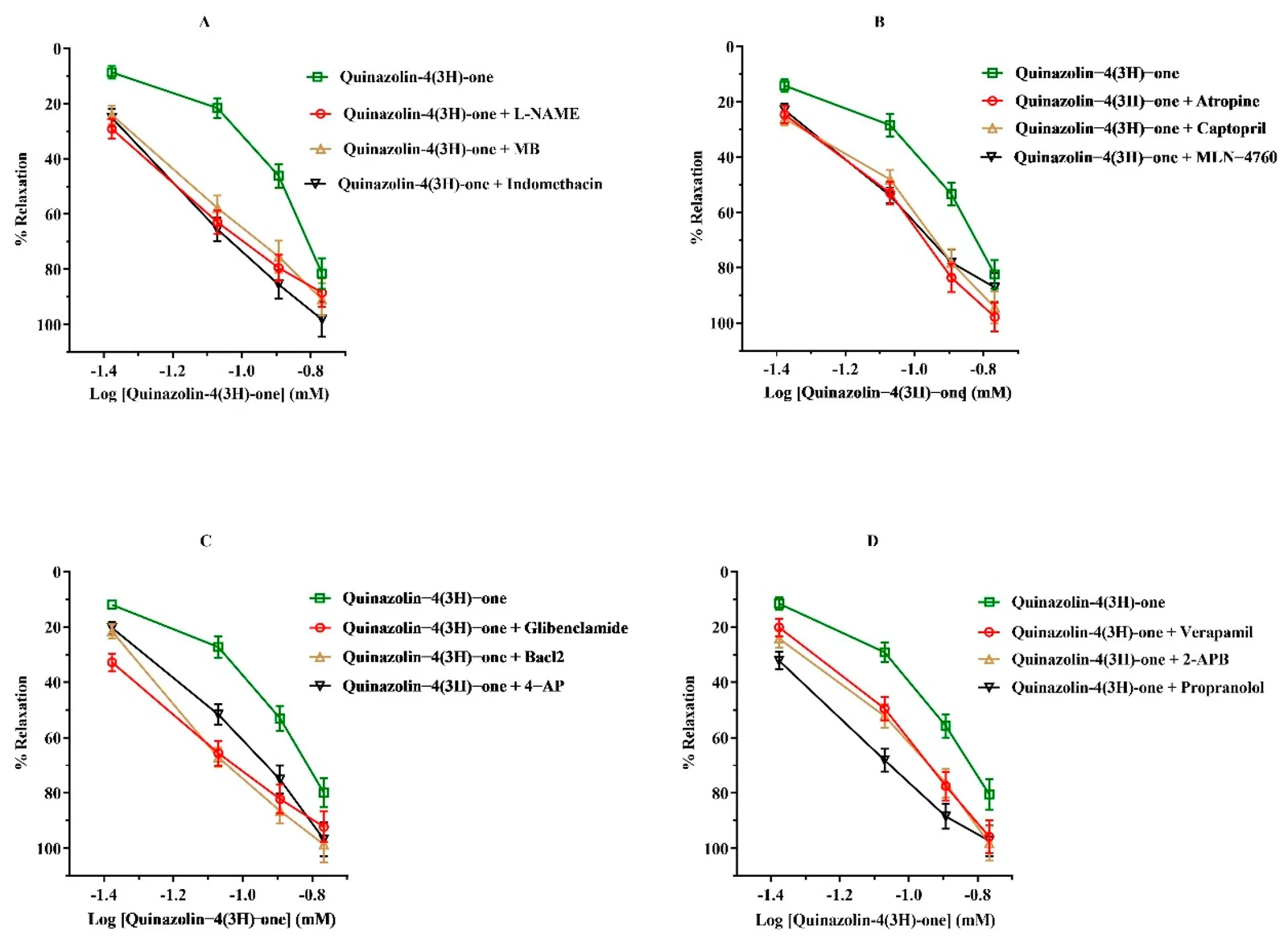
Aortic rings were exposed to various concentrations of quinazolin-4(3*H*)-one (0.042, 0.085, 0.128 and 0.171 mM) in the absence or presence of pre-incubation with inhibitors. The inhibitors tested were as follows: L-NAME, Methylene blue, and Indomethacin (**A**); Atropine, Captopril, and MLN-4760 (**B**); Glibenclamide, BaCl_2_, and 4-AP (**C**); and Verapamil, 2-Aminoethoxydiphenyl borinate (2-APB), and Propranolol (**D**). A sample size of *n* = 6 was used for all groups, with data shown as mean ± SEM compared to the untreated control.

Before EP (10 µM)-induced pre-contraction, endothelium-intact aortic rings were pre-incubated for 20 min with one of the following agents: L-NAME (10^−4^ M), methylene blue (10^−5^ M), indomethacin (10^−5^ M), atropine (10^−5^ M), captopril (10^−5^ M), MLN-4760 (6 nM), glibenclamide (10^−5^ M), BaCl_2_ (10^−4^ M), 4-AP (10^−4^ M), verapamil (10^−5^ M), 2-aminoethoxydiphenyl borinate (2-APB, 10^−4^ M), or propranolol (10^−5^ M). Pre-incubation with these agents resulted in numerically greater relaxation at some concentrations in several groups compared with quinazolin-4(3*H*)-one alone (Figure 3). However, these apparent differences did not reach statistical significance in the overall comparisons. Therefore, they cannot be considered evidence of a true pharmacological potentiation of the vasorelaxant effect. Rather, the present findings indicate that pre-incubation with the tested inhibitors/antagonists did not significantly attenuate the vasorelaxant response to quinazolin-4(3*H*)-one under the experimental conditions used.

#### 3.2.3. Effects of Quinazolin-4(3*H*)-one on PE-Induced Contraction in Isolated Rat Aortic Rings

**Figure 4:**
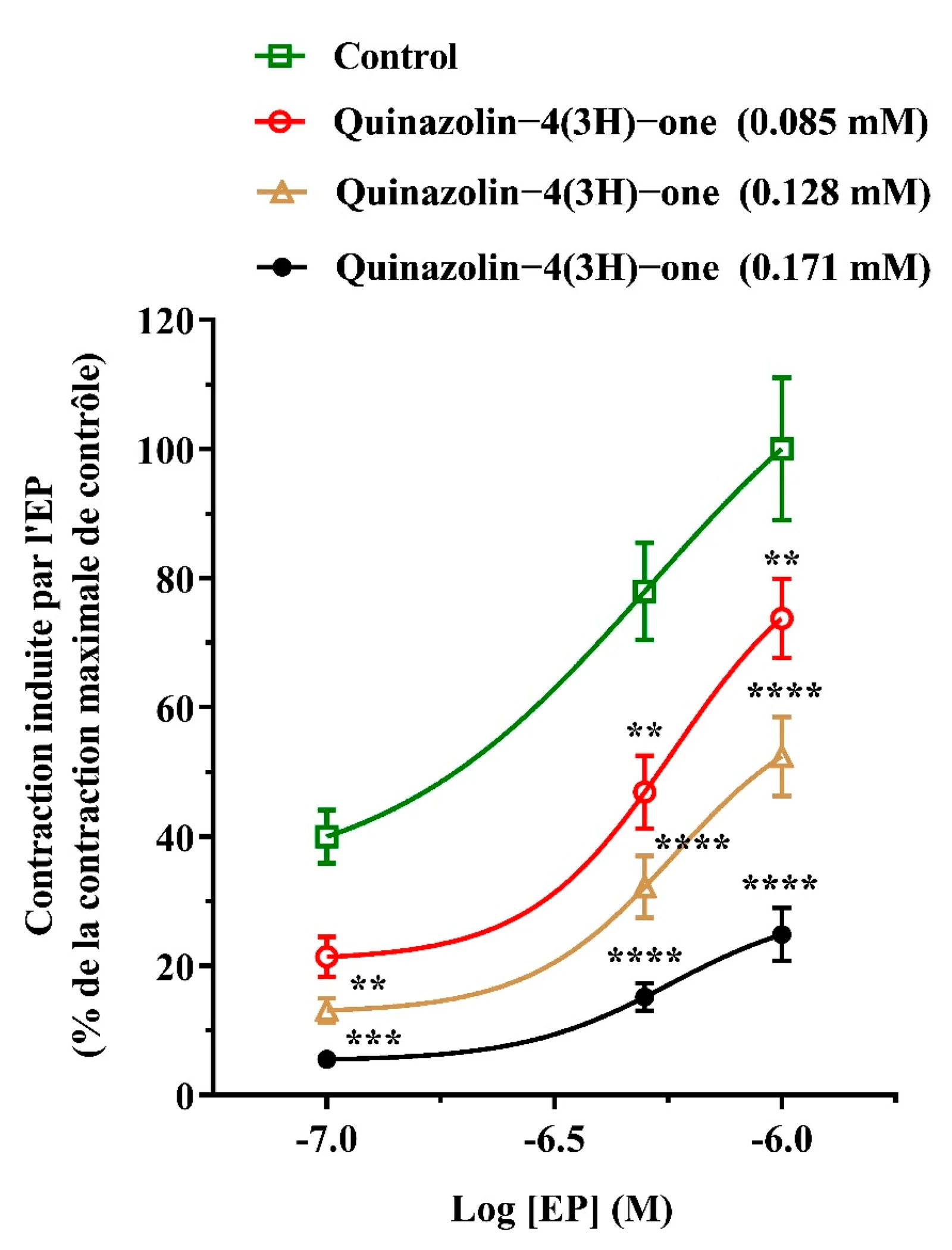
Effect of quinazolin-4(3*H*)-one on epinephrine (EP)-induced contraction of isolated rat thoracic aortic rings. Concentration-response curves to EP were obtained in the absence (Control) or presence of quinazolin-4(3*H*)-one (0.085, 0.128, and 0.171 mM). Contractile responses were normalized to the maximal EP-induced contraction in the control group and expressed as a percentage of the control maximum. Data reflect mean ± SEM (*n* = 6). ** *p* < 0.01, *** *p* < 0.001, **** *p* < 0.0001 vs. control.

As shown in Figure 4, quinazolin-4(3*H*)-one significantly inhibited EP-induced contractions in rat aortic rings in a concentration-dependent manner. Two-way ANOVA revealed a significant effect of concentration [F(2, 24) = 57.25, *p* < 0.0001, partial η^2^ = 0.827] and treatment group [F(3, 24) = 55.51, *p* < 0.0001, partial η^2^ = 0.874]. A significant concentration × treatment interaction was also observed [F(6, 24) = 2.662, *p* = 0.0401, partial η^2^ = 0.400], indicating that the effect of quinazolin-4(3*H*)-one varied significantly across the experimental conditions. Bonferroni’s multiple-comparisons test showed that, at the first concentration, the responses to quinazolin-4(3*H*)-one at 0.085, 0.128, and 0.171 mM differed significantly from the control, with adjusted *p*-values of 0.0861, 0.0078, and 0.0007, respectively. At the second concentration, the corresponding adjusted *p*-values were 0.0021, <0.0001, and <0.0001, respectively. At the third concentration, the adjusted *p*-values were 0.0096, <0.0001, and <0.0001, respectively.

### 3.3. Molecular Docking Study

**Figure 5:**
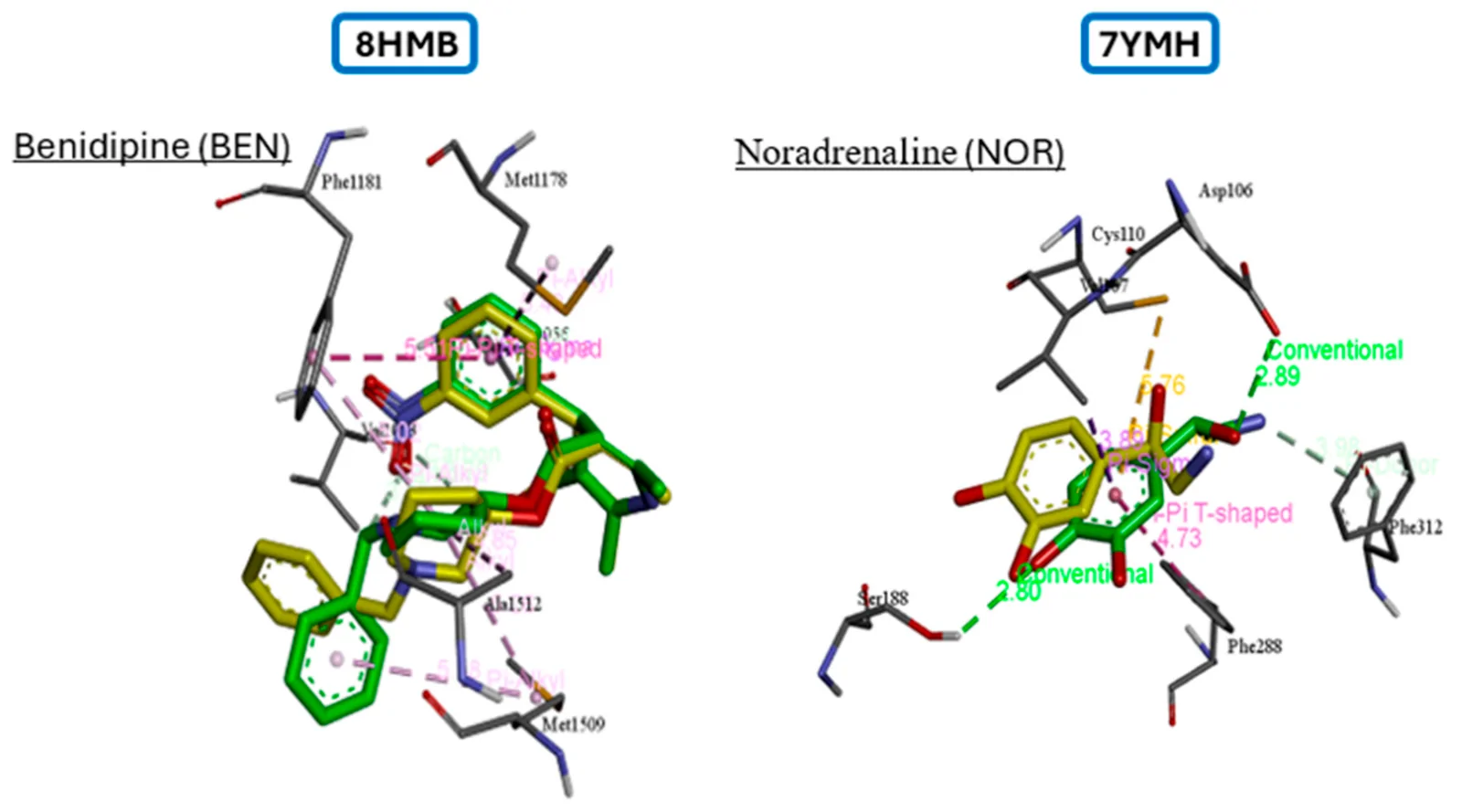
Validation of the docking method by redocking co-crystallised ligands. The native (green) and predicted (yellow) structures are displayed superimposed in the binding pockets of 8HMB and 7YMH.

Before proceeding with the actual docking experiment, a crucial step was undertaken in which redocking was performed for the co-crystallized ligands Benidipine and Noradrenaline in proteins 8HMB and 7YMH, respectively. During the redocking experiment, it was found that the RMSD values were 1.18 Å and 1.86 Å, respectively, both of which were within the generally accepted threshold of 2.0 Å, supporting the reliability of the docking protocol (Figure 5).

The results of the molecular docking study have provided particularly promising prospects. This analysis has made it possible to explore the binding affinities and modes of interaction of the novel quinazolin-4(3*H*)-one molecule with two major protein targets involved in vasorelaxation: the adrenergic α1-AR receptor and the L-type calcium channel (CaV1.2). The voltage-dependent L-type calcium channel CaV1.2 directly controls smooth muscle contraction, which is crucial for vascular relaxation. Verapamil, a well-known antagonist of L-type calcium channels, and terazosin, a particular antagonist of α1-adrenergic receptors, were used as reference drugs to create a rigorous comparison framework. Through these comparisons, the potential efficacy of the novel compound can be assessed in relation to current therapeutic standards. These compounds served as benchmarks for evaluating the performance of the quinazolin-4(3*H*)-one molecule within the active sites of the studied targets. The binding affinity results (Table 2) indicate that the quinazolin-4(3*H*)-one molecule demonstrates slightly more binding affinities in comparison with the reference drugs. Against the CaV1.2 channel, quinazolin-4(3*H*)-one achieved a docking score of −5.8 kcal/mol, which shows a score lower than the score of −7.0 kcal/mol for the calcium channel blocker Verapamil. showed a predicted docking score of −6.7 kcal/mol, compared with −6.5 kcal/mol for terazosin. Although the score for quinazolin-4(3*H*)-one was marginally favorable, the small difference of 0.2 kcal/mol does not support a conclusion of stronger binding affinity. Therefore, these docking results were considered only as preliminary evidence of a potential interaction between quinazolin-4(3*H*)-one and α1-AR.

**Figure 6:**
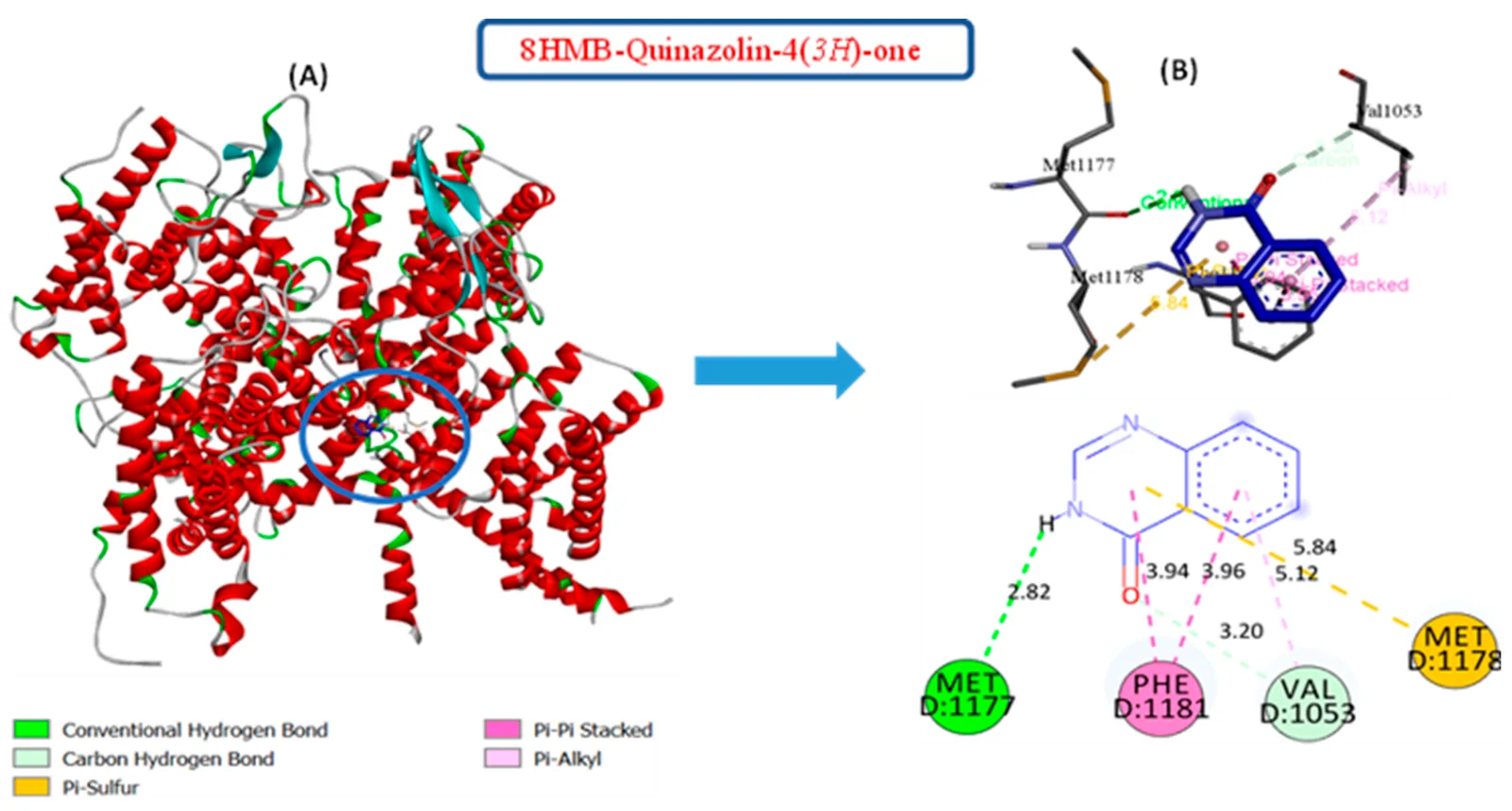
(**A**) Quinazolin-4(3*H*)-one molecule in the CaV.1.2 receptor pocket. (**B**) 3D and 2D views of interactions between Quinazolin-4(3*H*)-one molecule and the CaV.1.2 protein receptor (8HMB).

**Figure 7:**
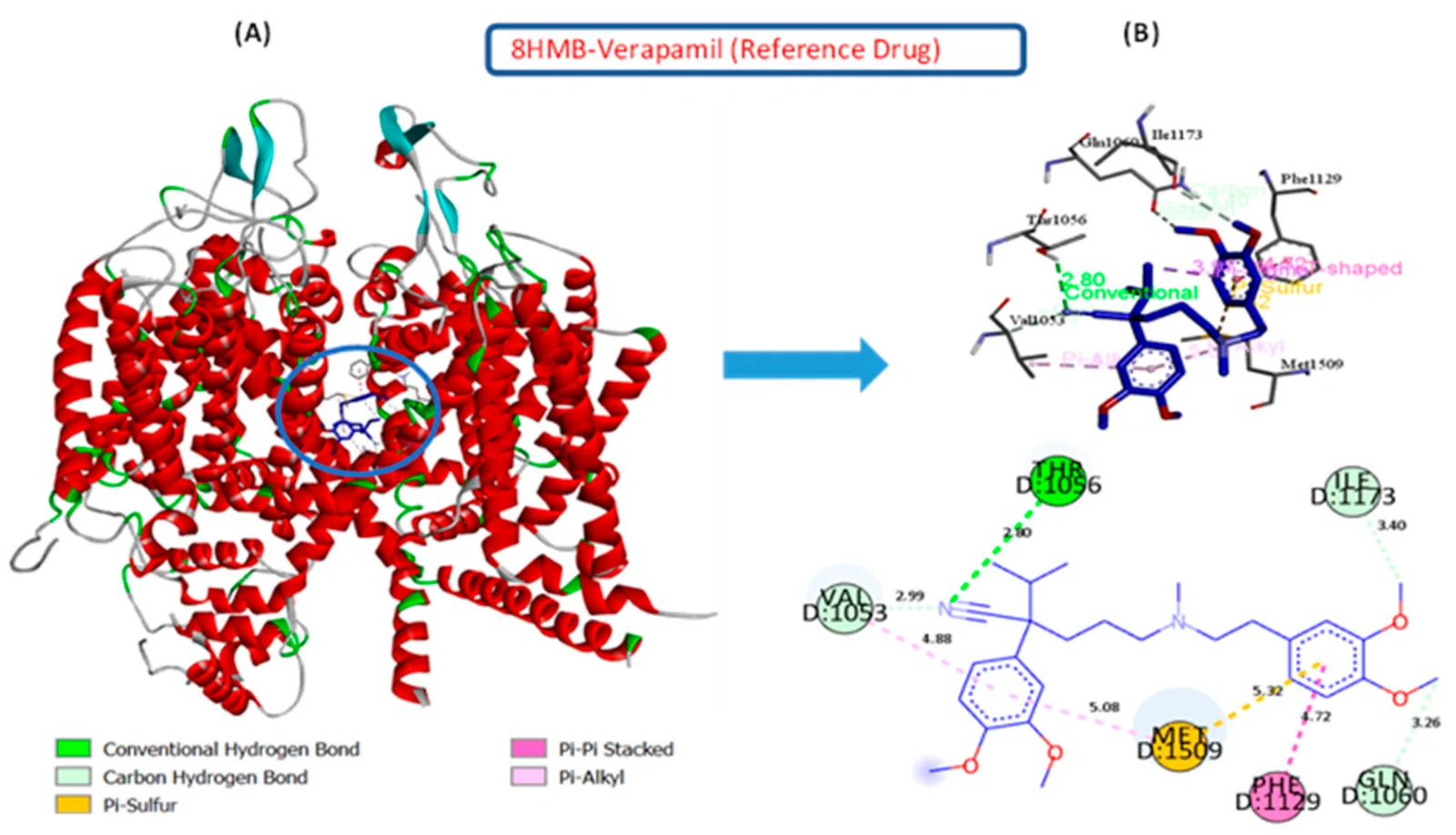
(**A**) Verapamil reference drug in the CaV.1.2 receptor pocket. (**B**) 3D and 2D views of interactions between Verapamil reference drug and the CaV.1.2 protein receptor (8HMB).

Upon examining the interactions resulting from molecular docking simulations, a detailed understanding of the binding modes within the active sites of the two targeted proteins was obtained. For the L-type calcium channel (CaV1.2), the 8HMB-Quinazolin-4(3*H*)-one complex demonstrated the formation of pivotal interactions, including a conventional hydrogen bond with the critical residue MET1177 (2.82 Å) and a carbon-hydrogen bond with VAL1053 (3.20 Å). Additionally, two hydrophobic pi-pi stacked interactions were observed with PHE1181 (3.94 Å), involving the aromatic rings of quinazolin-4(3*H*)-one and the carbon atoms of PHE1181’s aromatic ring. Additionally, a pi-sulfur interaction was observed with MET1178 (Figure 6). In contrast, the 8HMB-Verapamil complex showed a wider range of interaction types available, indicative of a greater array of binding modes. These interactions included a classical hydrogen bond, supplementary carbon-hydrogen bonds, hydrophobic pi-alkyl and pi-pi stacked interactions, and pi-sulfur interactions. The key residues participating in these interactions were THR1056 (2.80 Å), VAL1053 (2.99 Å), ILE1173 (3.40 Å), GLN1060 (3.26 Å), and MET1509 (5.32 Å) (Figure 7). This diversity in binding modes highlights the slight difference in the binding affinity value of Verapamil for the CaV1.2 active site compared to the quinazolin-4(3*H*)-one molecule.

**Figure 8:**
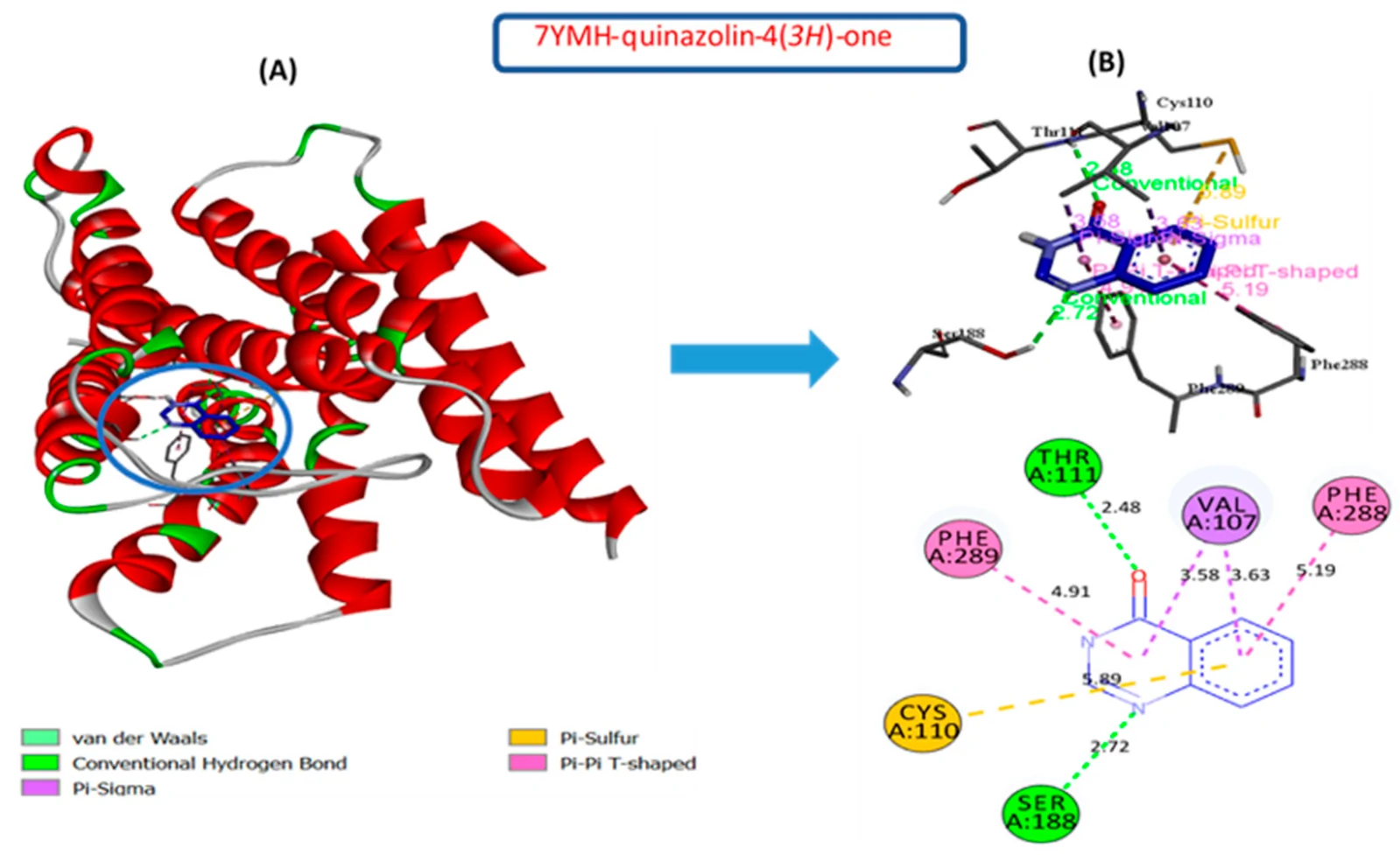
(**A**) quinazolin-4(3*H*)-one molecule in the α1-AR receptor pocket. (**B**) 3D and 2D views of interactions between quinazolin-4(3*H*)-one molecule and the α1-AR protein receptor (7YMH).

**Figure 9:**
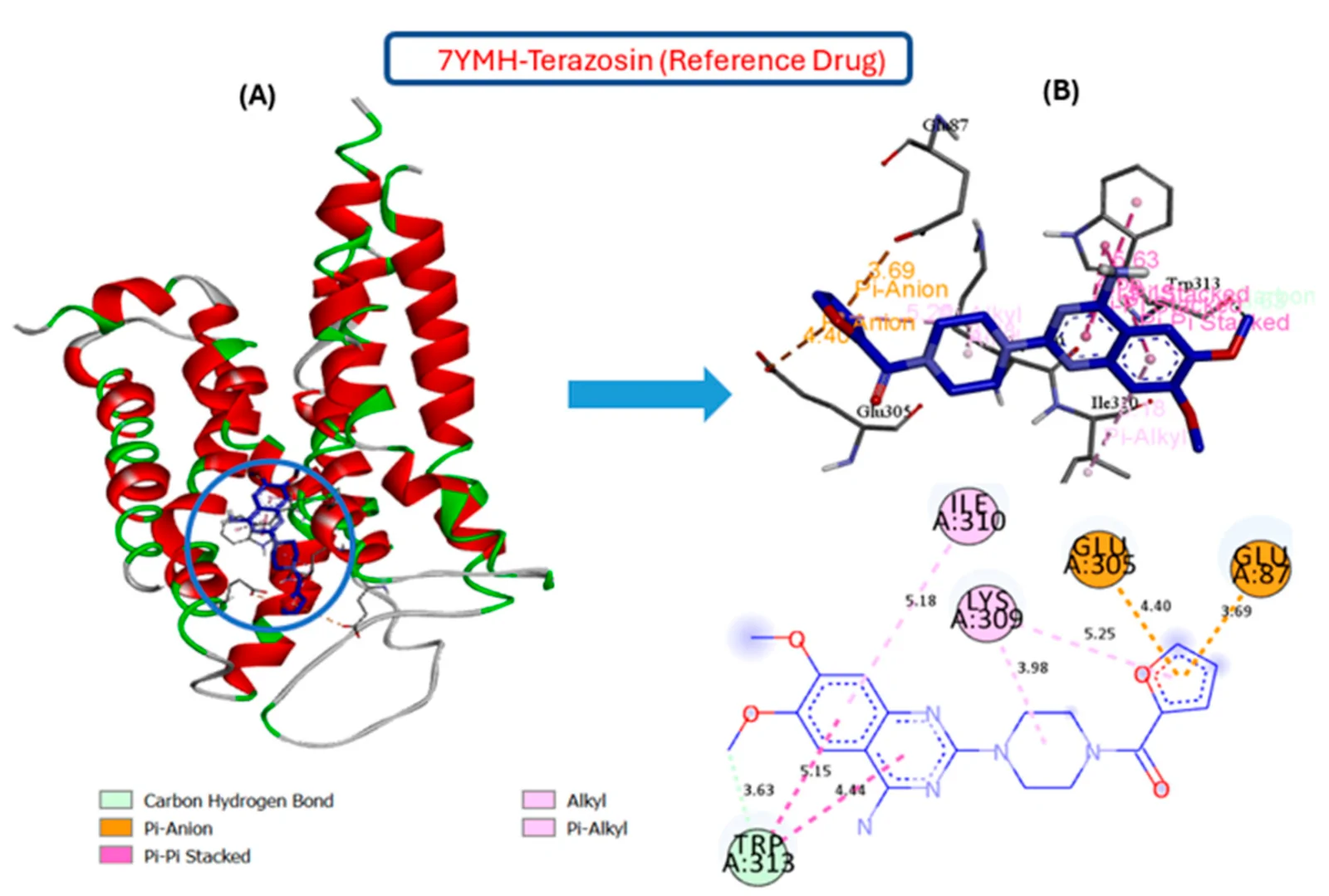
(**A**) Terazosin reference drug in the α1-AR receptor pocket. (**B**) 3D and 2D views of interactions between Terazosin reference drug and the α1-AR protein receptor (7YMH).

For the alpha-1 adrenergic receptor (α1-AR), the 7YMH-quinazolin-4(3*H*)-one complex exhibited significant molecular interactions that included conventional hydrogen bonds with THR111 (2.48 Å) and SER188 (2.72 Å). These bonds were established between the hydrogen atoms of the residues and the oxygen and nitrogen groups of the quinazolin-4(3*H*)-one molecules, which act as hydrogen bond acceptors. Furthermore, several hydrophobic interactions were identified, demonstrating the importance of non-covalent forces in stabilizing the complex. Two pi-pi stacked interactions with PHE288 (5.19 Å) and PHE289 (4.91 Å) enhanced ligand affinity by forming favorable aromatic stacking. In addition, two of the pi-sigma interactions between the carbon atoms of VAL107 (3.58 Å and 3.6 Å) and a couple of pi electrons in aromatic rings were also present in the ligand–protein docking complex. A pi-sulfur interaction, established between the quinazolin-4(3*H*)-one aromatic system and the sulphur-containing side chain of CYS110 (5.89 Å), demonstrates how specific interactions can help achieve optimized ligand-receptor recognition (Figure 8). Despite the presence of certain stabilizing characteristics, the 8HMB-terazosin complex exhibited a less diverse binding profile. A carbon-hydrogen bond was identified with TRP313 (3.63 Å), along with hydrophobic pi-pi stacked and pi-alkyl interactions involving TRP313 (4.44 Å and 5.15 Å), ILE310 (5.18 Å), and LYS309 (3.98 Å and 5.25 Å). A pi-anion interaction was also observed with GLU305 (4.40 Å) (Figure 9). These results suggest distinct interaction profiles for both complexes, with quinazolin-4(3*H*)-one potentially forming favorable contacts within the binding site.

### 3.4. Molecular Reactivity Analysis

DFT was employed to assess the key electronic parameters of the quinazolin-4(3*H*)-one molecule, and the results were compared to those of the reference drugs, Verapamil and Terazosin, in order to better understand their respective behaviors. The results of the DFT calculations are presented (Table 3). The energy value of the Highest Occupied Molecular Orbital (HOMO), which represents the ability of a molecule to donate electrons, is −6.6085 eV for quinazolin-4(3*H*)-one, which is less than that of Verapamil (−5.7497 eV) and Terazosin (−5.2855 eV), indicating greater electronic stability and a lesser ability to donate electrons. Alternatively, the energy value of the Lowest Unoccupied Molecular Orbital (LUMO), which represents the ability of a molecule to accept electrons, is −1.4310 eV for quinazolin-4(3*H*)-one. This value is moderate compared to Verapamil (−0.4337 eV) and Terazosin (−1.2024 eV). The energy gap (ΔE) of quinazolin-4(3*H*)-one is 5.1775 eV, which translates into slightly lower reactivity than that of Terazosin (4.0830 eV) and moderate chemical stability when compared to Verapamil (5.3160 eV). These figures corroborate the observations of the molecular docking study, where quinazolin-4(3*H*)-one’s interaction scores were similar to those of the reference molecules, which confirmed a similar ability to bind to the target site. In addition, quinazolin-4(3*H*)-one has a softness (S) of 0.3862 eV^−1^, a value that is in line with that of Verapamil (0.3762 eV) but slightly less than that of Terazosin (0.4898 eV). The hardness of quinazolin-4(3*H*)-one, reaches 2.5887 eV, placing it greater than that of Terazosin (2.0415 eV), but less than that of Verapamil (2.6580 eV). In summary, these results indicate that quinazolin-4(3*H*)-one has a balanced electronic profile. This configuration offers interesting prospects for establishing effective interactions with biological targets, while maintaining a satisfactory level of chemical stability. The electronegativity (χ) of quinazolin-4(3*H*)-one (4.0198 eV) is higher than that of both reference drugs (Verapamil: 3.0917 eV; Terazosin: 3.2440 eV), indicating a greater ability to attract electrons, which is a favorable property for interactions with biological targets. The potential (μ) of quinazolin-4(3*H*)-one is less, suggesting enhanced stability during chemical interactions. Finally, the electrophilicity index (ω), which measures a molecule’s propensity to interact with electrophilic sites, is 3.1209 eV for quinazolin-4(3*H*)-one, a moderate value compared to the typical requirements for effective inhibitors. Although it is larger than that of Terazosin (2.5773 eV) and Verapamil (1.7981 eV), this value remains within an acceptable range, which suggests that quinazolin-4(3*H*)-one has balanced electrophilic potential.

**Figure 10:**
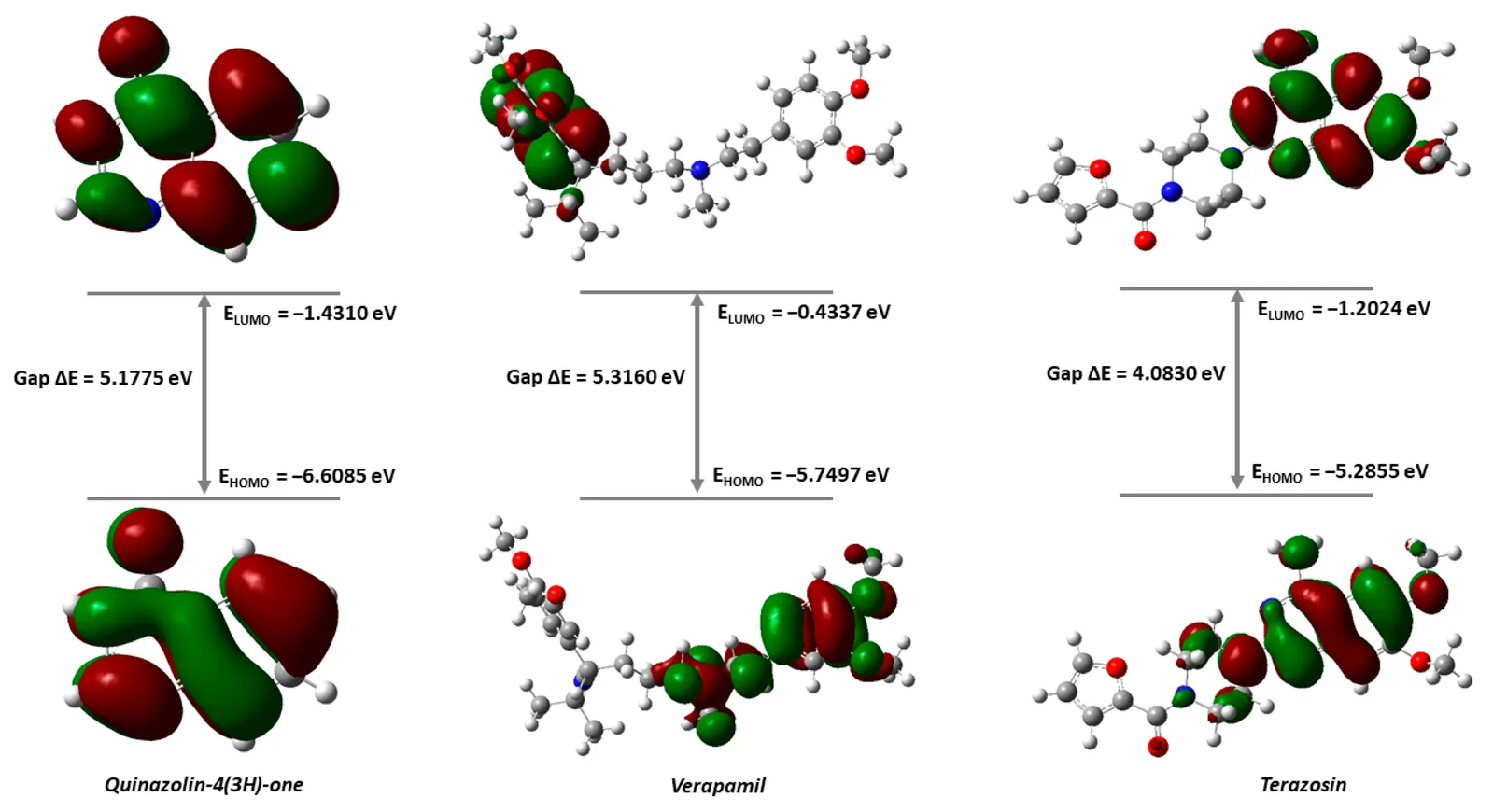
Spatial localization of HOMO and LUMO surfaces for quinazolin-4(3*H*)-one and standard reference compounds.

The localized HOMO-LUMO energy states for quinazolin-4(3*H*)-one and the reference drugs (Figure 10) suggest that the electronic density of the molecular orbitals is predominantly localized on the aromatic rings of quinazolin-4(3*H*)-one, indicating a strong involvement of these regions in interactions with biological targets. For Verapamil, the HOMO density is primarily localized on the methoxyphenyl groups as well as the central structure. Meanwhile, the LUMO is exclusively distributed over the methoxyphenyl groups, suggesting increased reactivity at potential intermolecular bonding sites. This distribution may explain Verapamil’s ability to form stabilizing interactions with various enzymatic residues. In the case of Terazosin, the HOMO-LUMO distribution shows a more balanced spread across the entire molecule, with significant concentration on the aromatic rings and amide groups, which could favor π-π interactions with aromatic residues of the targets. These observations confirm the importance of regions with high electronic density in molecular interactions and are consistent with the results of molecular docking simulations, where correlations between these regions and interaction residues were observed.

### 3.5. MEP Analysis and Geometric Optimization

**Figure 11:**
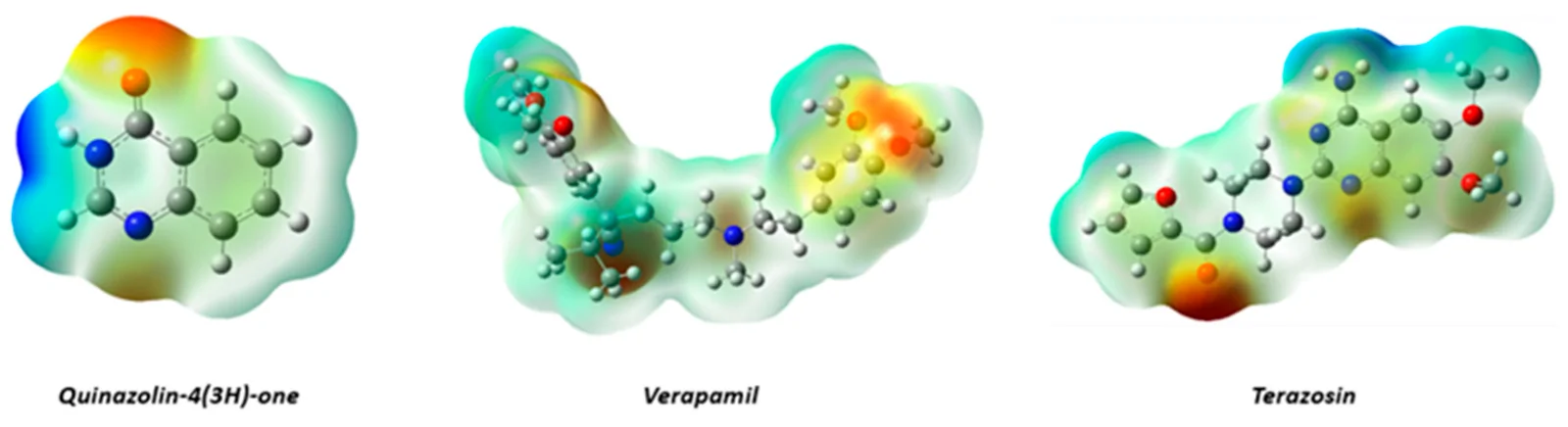
MEP plots of quinazolin-4(3*H*)-one and reference drugs.

Molecular electrostatic potential (MEP) maps for quinazolin-4(3*H*)-one, together with those for the reference drugs verapamil and terazosin, reveal a heterogeneous distribution of electrostatic potentials (Figure 11). The red and orange areas, which are abundant near nitrogen and oxygen atoms, indicate high electron density. Thus, these regions are recognized as most favored targets for electrophilic assaults. In contrast, blue areas primarily around hydrogen atoms indicate low electron density conducive to nucleophilic interactions. The green and yellow regions are neutral potential areas, observed almost exclusively along the carbon backbone.

### 3.6. Drug-likeness and ADME-Tox Evaluation

When the drug-likeness properties and pharmacokinetic profile of quinazolin-4(3*H*)-one, were probed by use of Lipinski’s rule of five, which is considered one of the most valuable heuristics for quantifying how many orally active drugs are among those known biomedical candidate compounds (Table 4), quinazolin-4(3*H*)-one meets the necessary characteristics. For instance, having a molecular mass <500 Daltons and ≤5 hydrogen bond donors and ≤10 hydrogen bond acceptors, and Log *p* < 5 without any rule of five violations. These results demonstrate the well-balanced polar and nonpolar properties of the molecule essential for oral absorption efficiency. Furthermore, the absence of PAINS alerts (0 alerts), a crucial sign of the authenticity of biological activity, implies that the biological impacts are real and not caused by structural artifacts. Finally, the calculated bioavailability score of 0.55 indicates a moderate probability of achieving adequate oral bioavailability.

The pharmacokinetic profile and safety of quinazolin-4(3*H*)-one were computationally predicted (Table 5). The predicted water solubility value of −0.984, in terms of absorption, is expected to play an important role in the future bioavailability of the compound. The predicted Caco-2 permeability, of 1.19, is satisfactory, indicating the molecule’s real potential to pass through the intestine and gain entry into circulation. The intestinal absorption rate of 74.394% in humans further supports this potential. This absorption rate indicates sufficient bioavailability, which is advantageous in avoiding the problems of absorption commonly encountered in therapy. With respect to the distribution, the volume of distribution at steady state (VDss), as calculated, reveals a negative logarithmic value of −0.332. This indicates the molecule’s tendency to stay within the vascular compartment, thus maximizing the effect on circulation. However, the chances of entry into the central nervous system are indicated through the negative log BB value of −0.197, obtained through blood–brain barrier permeability. This limited diffusion reduces the possibility of unfavorable neurological side effects. Quinazolin-4(3*H*)-one has a promising safety profile from a toxicological standpoint. It does not show any signs of hepatotoxicity or inhibition of hERG channels (types I or II), which are common indicators of cardiotoxicity, nor does it show any mutagenic potential, which is a crucial factor for long-term use. The molecule’s safety is further suggested the lack of any potential for skin sensitization. These results may suggest a favorable predicted safety profile, which warrants further experimental validation.

## 4. Discussion

The in vitro study evaluated the vasorelaxant activity of quinazolin-4(3*H*)-one in isolated aortic rings from Wistar rats and investigated the potential mechanisms underlying this effect. Various reference drugs were used to investigate the pathways involved in the vasorelaxant effect of quinazolin-4(3*H*)-one. The results indicated that, following pre-contraction induced by EP on intact and endothelium-denuded aortic rings isolated from rats, quinazolin-4(3*H*)-one produced a concentration-dependent vasorelaxant effect. Furthermore, endothelium removal did not reduce this effect, suggesting that the vasorelaxant activity of quinazolin-4(3*H*)-one is independent of the endothelium. Consequently, its mechanism might not be dependent on the vascular endothelium’s production of endothelium-derived relaxing factor (EDRF). Furthermore, KCl-pre-contracted aortic rings showed no significant relaxation compared to the control, suggesting that activation of voltage-dependent calcium channels is unlikely to be the primary mechanism underlying the vasorelaxant effect of quinazolin-4(3*H*)-one under the present experimental conditions. The present study demonstrated that pre-incubation with Atropine, MLN-4760, Captopril, L-NAME, and Indomethacin had no significant impact on the vasorelaxant effect of the quinazolin-4(3*H*)-one, confirming its endothelial-independent mechanism. These findings are consistent with those reported in previous studies [26,27,28]. The lack of significant attenuation of quinazolin-4(3*H*)-one-induced vasorelaxation by the twelve pharmacological inhibitors tested is an important finding. Under the present experimental conditions, these results argue against a major contribution of the conventional pathways targeted by these inhibitors, including muscarinic receptors, ACE/ACE2, NO/cGMP and prostaglandin pathways, β-adrenergic receptors, L-type calcium channels, store-operated Ca^2+^ entry, SERCA-dependent Ca^2+^ handling, and the K^+^ channels investigated (KATP, Kv, and Kir). However, the absence of an inhibitory effect should not be interpreted as definitive exclusion of these pathways, given the limitations inherent to pharmacological inhibition, including inhibitor selectivity and experimental conditions. Moreover, the active concentration range of quinazolin-4(3*H*)-one (0.042–0.171 mM) is relatively high and may be compatible with non-specific pharmacological effects. Therefore, the present inhibitor-screening results should be considered preliminary and hypothesis-generating, and further studies using more selective pharmacological and/or molecular approaches are required to identify the mechanisms underlying the vasorelaxant activity of quinazolin-4(3*H*)-one.

To investigate whether the vasorelaxant effect of quinazolin-4(3*H*)-one is associated with inhibition of the EP-induced contractile response, its effects were evaluated on EP-induced contractions in rat aortic rings. Importantly, EP is a non-selective adrenergic agonist with activity at both α- and β-adrenergic receptors [29]; inhibition of EP-induced contraction may reflect interference with adrenergic signaling and/or downstream mechanisms involved in vascular smooth muscle contraction. EP is a non-selective adrenergic agonist that activates both α- and β-adrenergic receptors; therefore, EP-induced contraction cannot be attributed exclusively to the activation of α_1_-adrenergic receptors. In the present study, the effects of quinazolin-4(3*H*)-one (0.085, 0.128, and 0.171 mM) were therefore evaluated on aortic rings pre-contracted with EP (10 μM) to determine whether this compound could interfere with adrenergic contractile responses. EP-induced vascular contraction primarily involves the activation of α-adrenergic receptors, which promotes Ca^2+^ mobilization through receptor-operated calcium channels (ROCCs) and the release of Ca^2+^ from intracellular stores, thereby increasing intracellular Ca^2+^ concentrations and promoting vascular smooth muscle contraction [30,31]. The results demonstrated that sustained contractions induced by cumulative concentrations of EP in rat aortic rings were significantly and dose-dependently attenuated by pre-incubation with quinazolin-4(3*H*)-one (0.085, 0.128, and 0.171 mM). These findings indicate that quinazolin-4(3*H*)-one interferes with the contractile response elicited by EP. However, the present findings do not provide sufficient evidence to establish a specific α1-adrenergic receptor-mediated mechanism. Thus, the observed inhibitory effect may result from modulation of adrenergic receptor-mediated signaling and/or downstream pathways involved in vascular smooth muscle contraction. These findings are consistent with previous reports showing that quinazolinone derivatives can attenuate adrenergic receptor-mediated vasoconstriction in rat aorta [32]. Several limitations of the present study should be acknowledged. First, although quinazolin-4(3*H*)-one attenuated EP-induced contractions, the use of a non-selective adrenergic agonist does not allow a specific attribution of this effect to α1-adrenergic receptors; therefore, experiments using selective α1-adrenergic agonists such as phenylephrine and receptor-binding approaches are needed. Second, the experiments were conducted exclusively in isolated rat aortic rings, and the vasorelaxant effects therefore remain to be validated in vivo. Third, the molecular docking findings provide predictive evidence of potential receptor interactions but require experimental validation. Finally, the study was conducted in a single animal species and within a limited concentration range, which may limit the generalizability of the findings. Consequently, the inhibitory effect observed may be attributed either to antagonism of α1-adrenergic receptors or to modulation of downstream signaling mechanisms that contribute to vascular smooth muscle contraction. Therefore, additional studies, including receptor-binding assays, subtype-selective pharmacological investigations, and intracellular signaling analyses, will be required to confirm the precise mechanism responsible for the observed vasorelaxant effect. Alpha-1 adrenergic receptor (α1-AR) antagonists, or alpha-blockers, inhibit type 1 alpha-adrenergic receptors, preventing smooth muscle contraction. They are primarily used to treat hypertension and benign prostatic hypertrophy by reducing vascular resistance in arterioles, increasing venous capacitance, and lowering blood pressure [33]. α1-Adrenergic receptors (α1-ARs), which belong to the G-protein coupled receptor superfamily, mediate the physiological effects of the endogenous catecholamines norepinephrine and EP. α1-ARs are critical in the regulation of the sympathetic nervous system [34]. Chronic activation of the sympathetic nervous system and α1-AR plays a significant role in the development of hypertension. Among the three subtypes of α1-AR-α1A, α1B, and α1D-α1D-AR is particularly critical for adrenergic responses in blood vessels such as the aorta, iliac, and mesenteric arteries. This subtype is closely associated with the regulation of normal and hypertension-related blood pressure. Current α1-AR blockers primarily target α1B-AR and are effective in lowering blood pressure; however, they are considered second-line treatments due to their potential adverse effects on cardiac and cerebrovascular outcomes. Therefore, selective targeting of α1D-AR may represent a potential therapeutic strategy for promoting vascular relaxation and managing hypertension, potentially with fewer side effects [35].

Due to its ability to regulate muscle contractility, CaV1.2 is considered a crucial therapeutic target in the treatment of cardiovascular diseases [36]. The CaV1.2 receptor is an excellent candidate as a strategic tool in the management of hypertension, as the inhibition of this receptor is directly proportional to the lowering of blood pressure [37]. Nevertheless, the results of the study show that the vasorelaxation of quinazolin-4(3*H*)-one is probably not mediated by the L-type calcium channel inhibition pathway. the alpha 1 adrenergic receptor plays a critical role in blood pressure management. Quinazolin-4(3*H*)-one has been found to have a very favorable binding score for the target protein α1-AR, thus showing significant interaction with this target protein, as confirmed by binding affinity studies. These interactions are mediated by molecular docking simulations in the active region of the target protein. The compound has more robust and varied stabilizing interactions with the target protein compared to the reference drug. <u>However, these in silico findings should be interpreted as supportive evidence of a potential interaction rather than confirmation of α1-AR-mediated vasorelaxation, particularly because the functional experiments in the present study were performed using EP rather than a selective α1-adrenergic agonist. Density functional theory (DFT), which is a very powerful tool in the study of the fundamental quantum state of molecular compounds, is used to obtain in-depth knowledge about the electrical properties and reactivity of the compounds under study [38]. DFT tests reveal that quinazolin-4(3*H*)-one exhibits increased electrical stability and a reduced propensity to act as an electron donor. These results are consistent with the results of the molecular docking simulations, which showed that the interaction scores of quinazolin-4(3*H*)-one is equivalent to those of reference drugs, suggesting a similar ability to bind to the target site. Furthermore, an important feature that enhances quinazolin-4(3*H*)-one interactions with biological targets is its increased electronegativity (χ).

One of the primary methods for forecasting the chemical reactivity of a compound and understanding its biological interactions is the measurement of the molecular electrostatic potential. MEP plays a critical role in understanding the interactions that take place between molecules. Furthermore, it helps in locating the reactive points in the molecules, which can be depicted graphically as molecular electrostatic potential surfaces [39]. The analysis of quinazoline-4(3*H*)-one using the MEP method shows that there is a unique distribution of charge between quinazolin-4(3*H*)-one and other pharmaceutical compounds, with electron-rich and electron-deficient areas that can affect their binding affinity to biological targets. It is interesting to note that the reactive sites obtained using the MEP method match the interaction sites obtained using the molecular docking approach, which confirms the potential of the compound. In order to filter compounds that can be used in medicine, it is important to determine their drug-like properties and pharmacokinetic parameters, including absorption, distribution, metabolism, excretion, and toxicity (ADME-Tox). These parameters are important in determining the oral bioavailability of compounds and establishing a direct relationship between their physicochemical properties and their biological activity in the organism [40]. In this context, the in silico evaluation of the above parameters is described as an indispensable step in the early stages of drug discovery, as it helps in the discovery of promising drug candidates while minimizing the time and costs involved in the conventional approach [41,42]. Quinazolin-4(3*H*)-one has good drug-like properties. The structure meets Lipinski’s rule of five with no PAINS alerts. In addition, the compound has excellent bioavailability. The intestinal absorption of the drug is high. The penetration of the drug into the central nervous system is low. As a result, there will be fewer central nervous system-related adverse effects. The metabolism and excretion of the drug are excellent. The drug does not have CYP inhibitors. In addition, the drug is not mutagenic, hepatotoxic, or cardiotoxic. From the above results, it can be concluded that the drug has excellent drug-likeness properties.

## 5. Conclusions

Quinazolin-4(3*H*)-one exhibited promising endothelium-independent vasorelaxant activity in isolated rat aortic rings and significantly inhibited EP-induced contraction. However, the present findings do not establish a specific α1-adrenergic receptor antagonistic mechanism. Molecular docking suggested a potential interaction with α1-AR, while DFT, MEP, and ADME-Tox analyses indicated favorable molecular and drug-like properties. Further pharmacological, in vivo, and toxicological studies are required to clarify the underlying mechanisms and evaluate its potential as a lead compound for antihypertensive drug development.

## Figures and Tables

**Table 1:** Active site coordinates of studied proteins (8HMB and 7YMH).

Protein	8HMB	7YMH
Size (Ǻ)	X = 10	X = 30
	Y = 10	Y = 30
	Z = 10	Z = 30
Center (Ǻ)	X = 167.02	X = 118.85
	Y = 164.68	Y = 121.96
	Z = 154.11	Z = 143.22

**Table 2:** The values of the binding affinity energy (docking scores) of the calculated molecular docking of quinazolin-4(3*H*)-one and reference drugs against the two targeted vasorelaxant activities, CaV1.2 (PDB ID: 8HMB) and α1-AR (PDB ID: 7YMH).

Docked Compounds	Docking Scores (Kcal/mol)		
	Quinazolin-4(3*H*)-one	Verapamil	Terazosin
CaV1.2(8HMB)	−5.8	−7.0	-
α1-AR (7YMH)	−6.7	-	−6.5

**Table 3:** Calculated frontier molecular orbital parameters of quinazolin-4(3*H*)-one and reference drugs.

Compound	HOMO (eV)	LUMO (eV)	Gap ∆E	IP	EA	χ	µ	ƞ	S	ꞷ
Quinazolin-4(3*H*)-one	−6.6085	−1.4310	5.1775	6.6085	1.4310	4.0198	−4.0198	2.5887	0.3862	3.1209
Verapamil	−5.7497	−0.4337	5.3160	5.7497	0.4337	3.0917	−3.0917	2.6580	0.3762	1.7981
Terazosin	−5.2855	−1.2024	4.0830	5.2855	1.2024	3.2440	−3.2440	2.0415	0.4898	2.5773

Gap ∆E: LUMO–HOMO; IP (−HOMO): ionization potential; EA (−LUMO): electron affinity; χ(IP + EA)/2: electronegativity; µ − (IP + EA)/2: chemical potential; ƞ (IP − EA/)2: chemical hardness; S (1/ƞ): chemical softness; ꞷ (µ^2^/2ƞ) electrophilic index.

**Table 4:** In silico drug-likeness assessment of quinazolin-4(3*H*)-one: Compliance with Lipinski’s criteria.

Compound	Drug Likeness Properties
	Quinazolin-4(3*H*)-one
MW (g/mol)	146.15
H-Bonds Acceptors	2
H-Bonds Donors	1
Log *p*	1.21
Rotatable Bonds (Flexibility)	0
Lipinski Violation	0
PAINS Alerts	0
Bioavailability Score	0.55

**Table 5:** Predicted ADME-Tox properties for quinazolin-4(3*H*)-one.

	Properties	Compound
ADMET		Quinazolin-4(3*H*)-one
Absorption	Water solubility (log mol/L)	−0.984
	Caco2 permeability (log Papp in 10^−6^ cm/s)	1.19
	Intestinal absorption (human)%	74.394
Distribution	VDSS (Human) (log L/kg)	−0.332
	BBB Permeability (log BB)	−0.197
	CNS Permeability (log PS)	−1.938
Metabolism	CYP1A2 inhibitor	Yes
	CYP2C19 inhibitor	No
	CYP2C9 inhibitor	No
	CYP2D6 inhibitor	No
	CYP3A4 inhibitor	No
Excretion	Total clearance (log mL/min/kg)	0.635
Toxicity	Mutagenicity	No
	hERG I inhibitor	No
	hERG II inhibitor	No
	Hepatotoxicity	No
	Skin sensitization	No

## Data Availability

The original contributions presented in this study are included in the article/Supplementary Materials. Further inquiries can be directed to the corresponding authors.

## Supplementary Materials

The following supporting information can be downloaded at https://www.mdpi.com/article/10.3390/cimb48090963/s1.
