# Supplementary material for: In Vitro Experimental and Computational Studies of the Vasorelaxant Effects of Quinazolin-4(3H)-one and Its Interaction with α1-Adrenergic Receptors
Source: Curr Issues Mol Biol. 2026 Sep 20;48(9):963. doi: 10.3390/cimb48090963 (PMC13605234; doi:10.3390/cimb48090963)
Supplement: Supplementary file 1 [file cimb-48-00963-s001.zip › cimb-4464058-supplementary.pdf]

Supplementary Materials

In Vitro Experimental and Computational Studies of the Vasorelaxant Effects of Quinazolin-4(3*H*)-one and Its Interaction with α1-Adrenergic Receptors

Yassine Rhazi [1,*], Ismail Bouadid [2], FatimaZahra Guerguer [3], Mohammed El mesky [4], Houda Lamssane[5], Asmae Nakkabi [4], Mohamed Bakhouch [6], Malak Yahia Qattan [7], Noorah A. Alkubaisi [8], Samir Chtita [3], Mourad A. M. Aboul-Soud [9,*], John P. Giesy [10,11,12], Mohamed El Yazidi [1], Mohamed Eddouks [2]

[1]Engineering Laboratory of Organometallic, Molecular and Environment, Faculty of Sciences Dhar EL Mahraz, Sidi Mohamed Ben Abdellah University, P.O. Box 1796, Atlas, Fez 30000, Morocco.

[2] Team of Ethnopharmacology and Pharmacognosy, Faculty of Sciences and Techniques Errachidia, Moulay Ismail University of Meknes, BP 509, Boutalamine, Errachidia, Morocco.

[3] Laboratory of Analytical and Molecular Chemistry, Faculty of Sciences Ben M'Sik, Hassan II University of Casablanca, Casablanca P.O. Box 7955, Morocco.

[4] Laboratory of Materials Engineering for the Environment and Natural Resources, Faculty of Sciences and Techniques, University of Moulay Ismail of Meknès, B.P 509, Boutalamine, Errachidia 52000, Morocco.

[5]Laboratory of Applied Organic Chemistry, Faculty of Science and Techniques, Sidi Mohamed Ben Abdellah University, Fez 30050, Morocco

[6] Laboratory of Bioorganic Chemistry, Department of Chemistry, Faculty of Sciences, Chouaïb Doukkali University, P.O. Box 24, El Jadida 24000, Morocco.

[7] Department of Health Sciences, College of Applied Studies, King Saud University, P.O. Box 4545, Riyadh 11451, Saudi Arabia.

[8] Department of Botany and Microbiology, College of Science, King Saud University, Riyadh 11451, Saudi Arabia.

[9] Center of Excellence in Biotechnology Research, College of Applied Medical Sciences, King Saud University, Riyadh 11433, Saudi Arabia.

[10] Department of Veterinary Biomedical Sciences and Toxicology Centre, Western College of Veterinary Medicine, University of Saskatchewan, Saskatoon, SK S7N 5B4, Canada.

[11] Department of Integrative Biology and Center for Integrative Toxicology, Michigan State University, East Lansing, MI 48824, USA.

[12] Department of Environmental Sciences, Baylor University, Waco, 76706, USA.

[*] Corresponding authors:

E-mail addresses: yassine.rhazi@usmba.ac.ma (Y.R) and maboulsoud@ksu.edu.sa

1. Materials and methods

a. Chemical reagents and drugs

Acetylcholine chloride, Epinephrine, N-nitro-L-arginine methyl ester (L-Name), Indomethacin, Methylene Blue, 2-Aminoethoxydiphenyl borinate (2-APB), 4-Aminopyridine, Barium chloride ($BaCl_2$) and MLN-4760 were obtained from Sigma Chemical Co. (St. Louis, USA). Atropine was sourced from Chem Cruz. Other reagents were of analytical grade and procured locally. All mentioned drugs were dissolved in distilled water.

b. Measurement of vascular relaxation and evaluation of mechanisms involved

Male rats, anesthetized with pentobarbital sodium (50 mg/kg) [1], are humanely sacrificed and subjected to detailed dissection procedures. The excision of the aorta is followed by immediate placement in a cold buffer to preserve tissue integrity. Meticulous cleaning removes any adhering fat and connective tissue, ensuring pure samples for study. The thoracic aorta is then precisely sectioned into 3–4 mm rings. These rings are suspended under a consistent resting force of 2 g within continuously aerated tissue bath containing 40 ml of Krebs-Henseleit solution and carbogen (95% O_2 and 5% CO_2). The solution is carefully maintained at 37°C and a pH of 7.4, with a specific composition of NaCl (118 mM), KCl (4.50 mM), $NaHCO_3$ (25 mM), $MgSO_4$ (1.2 mM), $CaCl_2$ (1.8 mM), $NaHPO_4$ (1.2 mM), and glucose (11 mM). recording changes in force isometrically using a lever transducer from Erma, Tokyo. Each aortic ring is mounted on stainless steel hooks one fixed to the chamber's base and the other attached to a UF1 force transducer from LCM Systems Ltd. This setup is connected to a PowerLab/400 ADInstrument data acquisition system, facilitating precise recording of isometric tension, a critical parameter for our vascular studies [2]. The study was carried out on aortic rings with intact endothelium. Endothelial integrity was confirmed if acetylcholine (ACh, 10^{-4} M) induced 40-60% relaxation of aortic rings precontracted by (EP) (10 μM) [2]. For the endothelium-denuded aortic rings, the endothelium was carefully removed by insertion a wooden tool into the lumen of the rings and gently rubbing the ring for a few seconds before placing the aorta into the tissue bath chamber. Successful removal of the endothelium was verified by observation of less than 15% relaxation in response to ACh (10^{-4} M) in EP-precontracted aortic rings [1], as described in previous studies with some modifications optimal tension, selected from preliminary experiments, was the tension which gave the greatest response to 10 μM Epinephrine (EP). The rings were given 2g (100%) of initial tension and allowed to equilibrate for one hour. Thirty minutes after setting up the tissue bath aortas rings were contracted with

10 μM EPor 80 mM potassium chloride (KCl) solution to test their contractile responses. Following exposure to EP or KCl, tissues were washed three times with Krebs solution to restore basal tension and this solution was renewed approximately every 60 min [3]. To determine the cumulative concentration-dependent curves for compound-induced relaxation and after equilibration rings were contracted by EP (10 μM). After the plateau was reached, quinazolin-4(*3H*)-one was added in increasing concentrations cumulatively (0.042, 0.085, 0.128 and 0.171 mM), the curve was allowed to reach a plateau before adding a subsequent concentration after 10 min. The following experiment was conducted on rat aortic rings pre-contracted by EP to identify the mechanistic pathway behind the relaxant effect indued by quinazolin-4(*3H*)-one: Before adding EP, aortic rings were pre-incubated for 20 minutes with one of many common drugs: 1) 10^{-4} M L-NAME, a direct inhibitor of nitric oxide (NO) Synthase. 2) 10^{-5} M Indomethacin, a prostaglandin synthesis inhibitor. 3) 10^{-5} M Captopril, angiotensin-converting enzyme inhibitors (ACE). 4) 6 nM MLN-4760 an ACE-2 inhibitor 5) Glibenclamide, an ATP-sensitive K^+ channel blocker. 6) 10^{-5} M Verapamil, a L-type calcium channel blocker. 7) 10^{-5} M Methylene blue (MB), a Guanylate cyclase inhibitor. 8) 10^{-5} M Propranolol, a beta-blocker. 9) 10^{-5} M Atropine a muscarinic receptor antagonist. 10) 10^{-4} M 2-aminoethoxydiphenyl borinate (2-APB), a blocker of store-operated Ca^{2+} entry; inositol 1,4,5-trisphosphate (InsP(3))-mediated Ca^{2+} release and an inhibitor of SERCA Ca^{2+} pumps. 11) 10^{-5} M Barium chloride ($BaCl_2$) an inhibitor of inwardly rectifying K^+ channels (a K_{IR} blocker). 12) 10^{-4} M 4- Aminopyridine (4-AP) a voltage-dependent K^+ channel inhibitor (a K^+_V blocker) [3]. The contraction percentage in response to EP was used to calculate the vasorelaxant impact on the aortic rings. The aortic ring was cleaned three times before any of the chemicals used in the experiment were changed (the aorta ring should be incubated for five minutes at a time in each bath). To serve as a control, vehicle (distilled water) was added cumulatively in volumes matching those of the Quinazolin-4(3H)-one solution to aortic rings pre-contracted with EP (10 μM) and KCl (80 mM) [4].

2. ^{1}H, ^{13}C NMR spectra of quinazolin-4(3*H*)-one

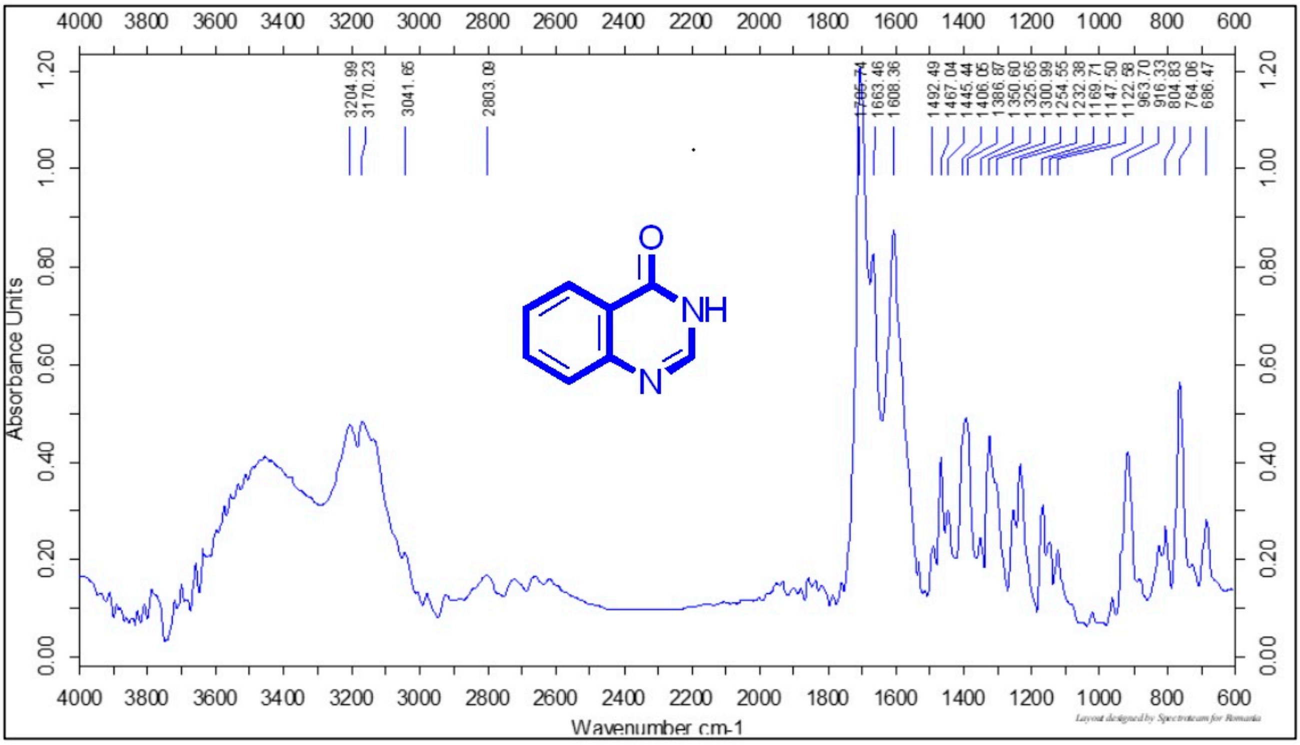

Figure S1. IR spectrum of *quinazolin-4(3H)-one*

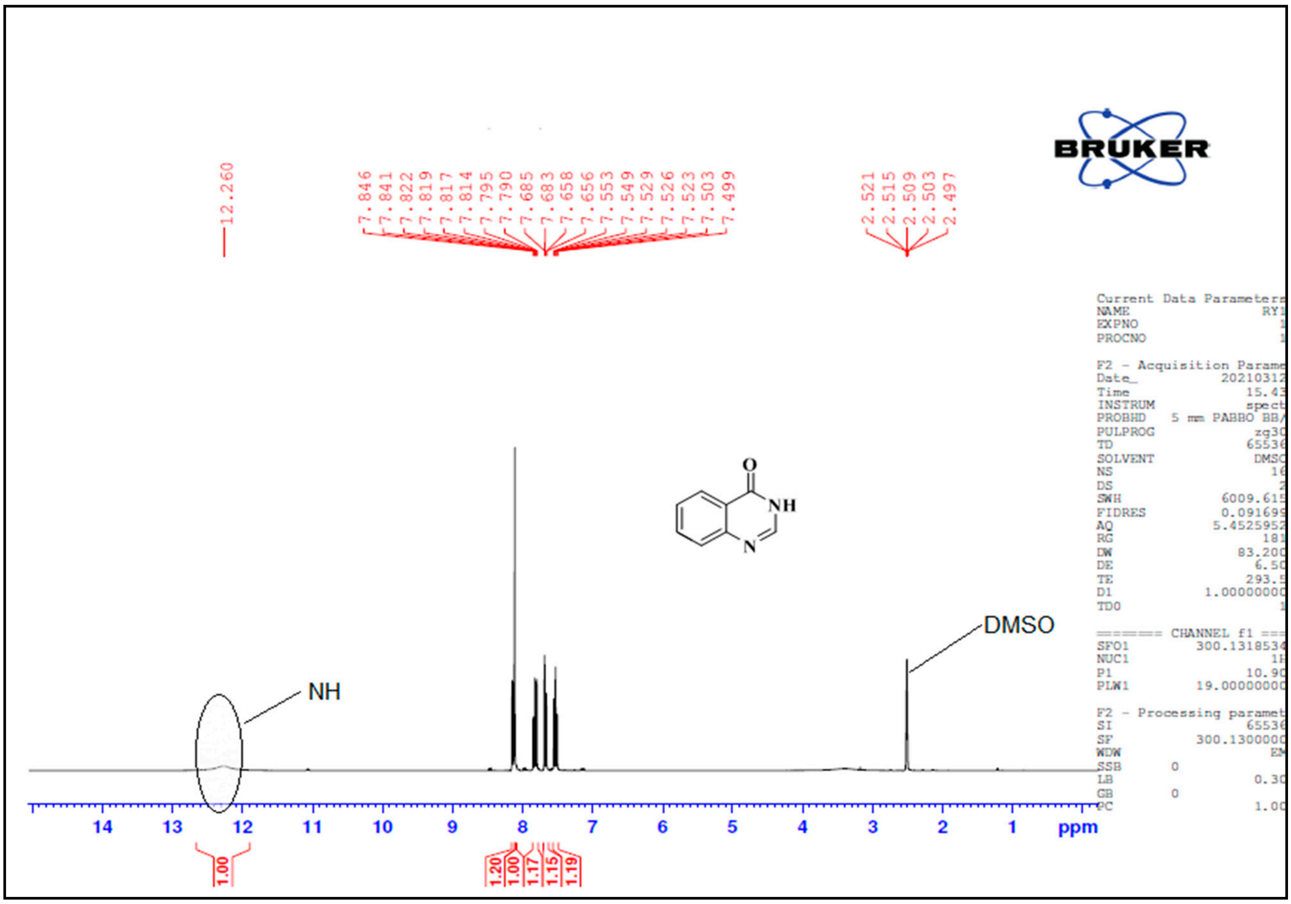

Figure S2. ^{1}H NMR spectrum (300 MHz, DMSO) of *quinazolin-4(3*H*)-one*

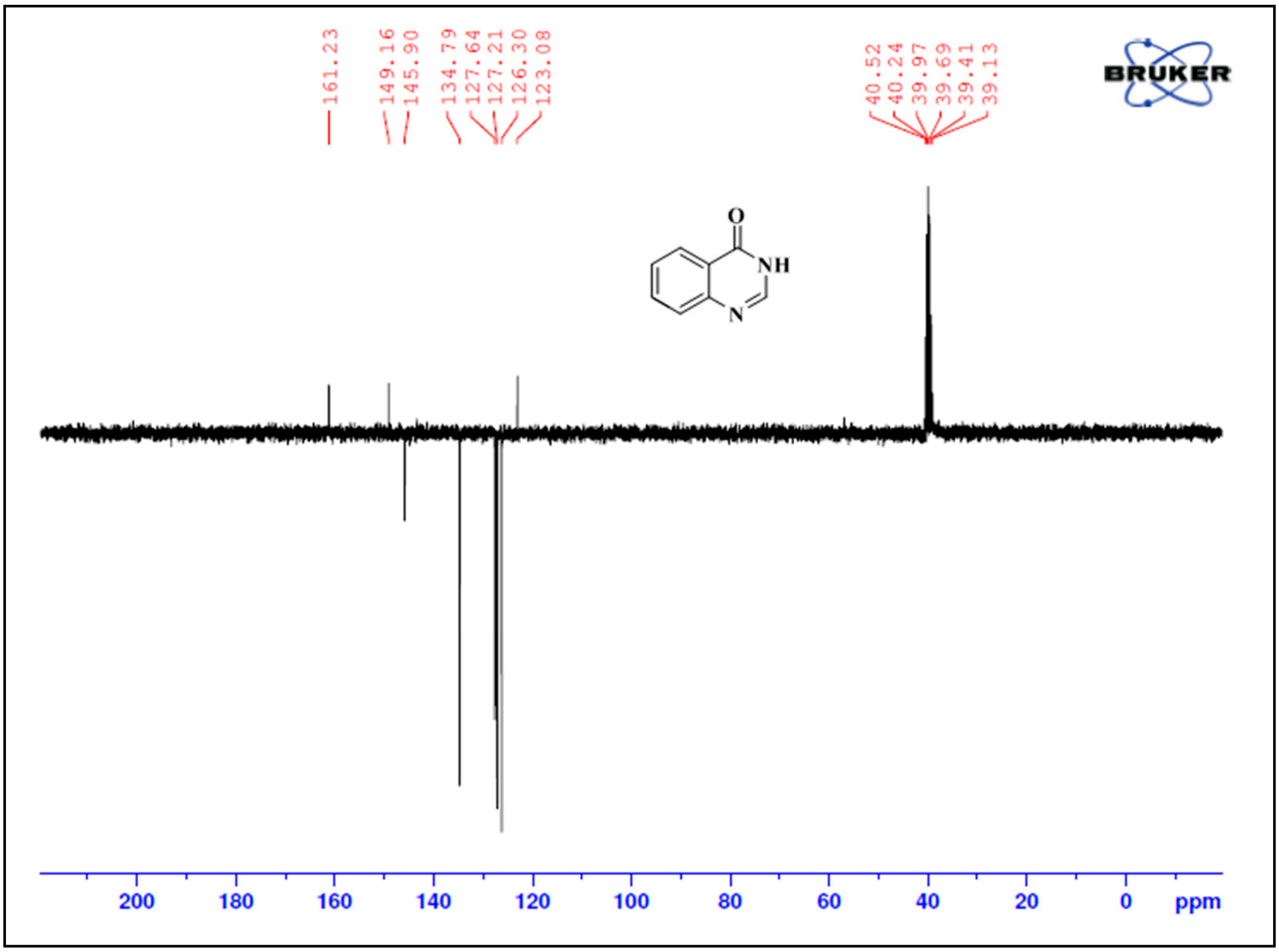

Figure S3. ^{13}C NMR spectrum (75 MHz, DMSO) of *quinazolin-4(3H)-one*

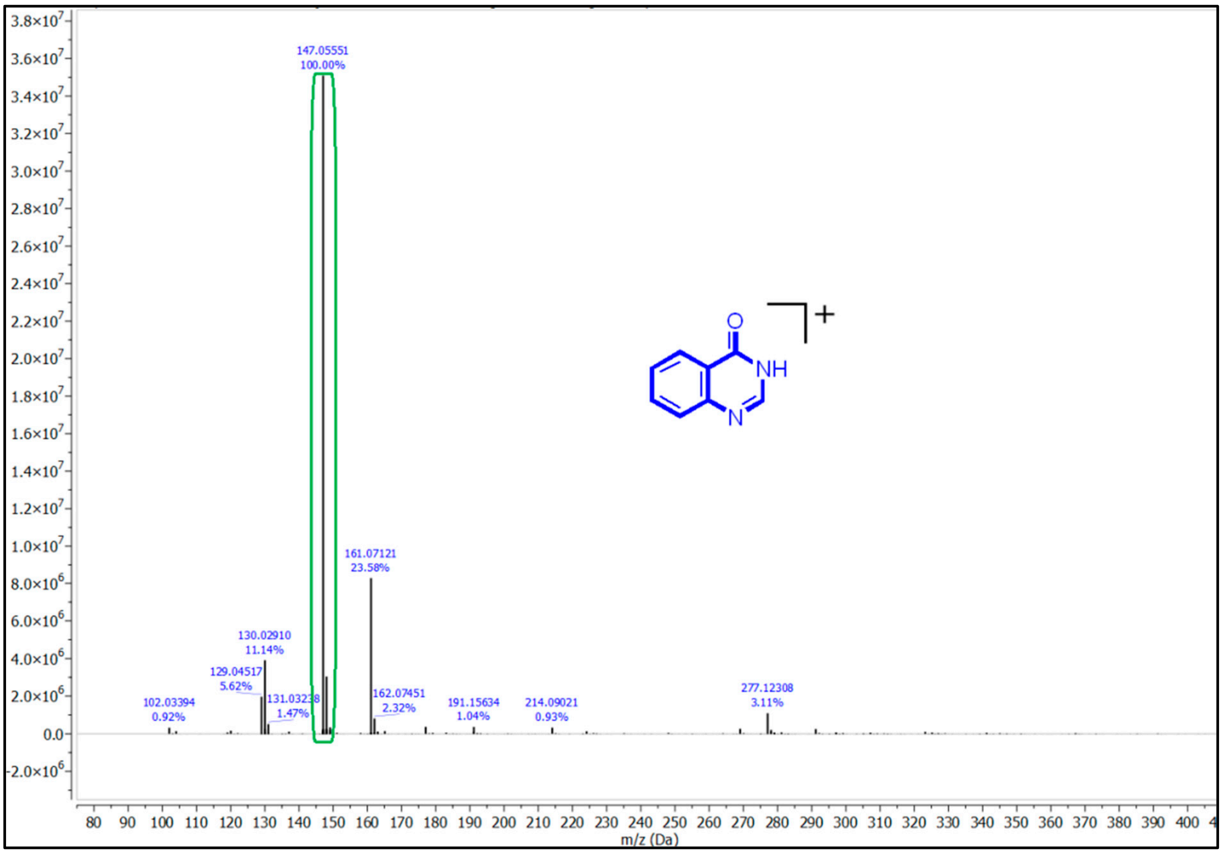

Figure S4. Mass spectrum of *quinazolin-4(3H)-one*

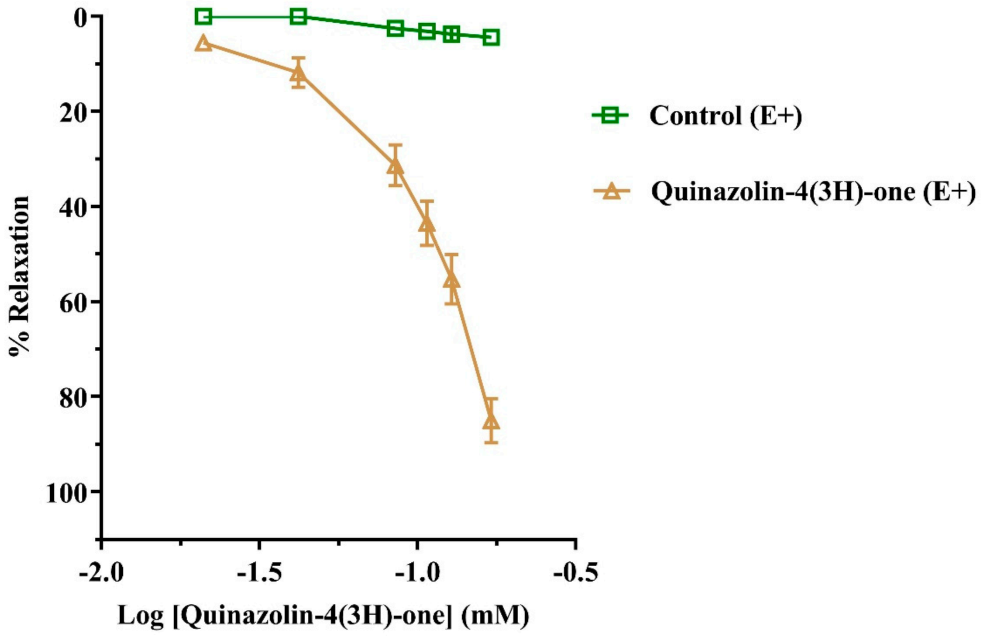

Figure S5. Preliminary concentration–response curve of quinazolin-4(3H)-one-induced vasorelaxation in rat thoracic aortic

References:

1. Ok, S.H.; Bae, S. Il; Shim, H.S.; Sohn, J.T. Dexmedetomidine-Induced Contraction of Isolated Rat Aorta Is Dependent on Extracellular Calcium Concentration. *synapse.koreamed.orgSH Ok, SI Bae, HS Shim, JT SohnKorean Journal of Anesthesiology, 2012•synapse.koreamed.org* **2012**, *63*, 253–259, doi:10.4097/KJAE.2012.63.3.253.

2. Ajebli, M.; Eddouks, M. Antihypertensive Activity of Petroselinum Crispum through Inhibition of Vascular Calcium Channels in Rats. *J. Ethnopharmacol.* **2019**, *242*, 112039, doi:10.1016/J.JEP.2019.112039.

3. Bouadid, I.; Qabouche, A.; Hebi, M.; El Mesky, M.; Alhaji, J.H.; Aboul-Soud, M.A.M.; Giesy, J.P.; Eddouks, M. Antihypertensive Effects of Benzaldehyde in Rats: Involvement of Endothelium-Independent and Endothelium-Dependent Vasorelaxation via Prostacyclin (PGI2)/CAMP Pathway Activation and Calcium Channel Inhibition. *Pharmaceuticals 2026, Vol. 19, Page 945* **2026**, *19*, 945, doi:10.3390/PH19060945.

4. Bouadid, I.; Qabouche, A.; Eddouks, M. Antihypertensive and ACE-2 Inhibitory Effects of Daphne Gnidium in Rats. *Cardiovasc. Hematol. Agents Med. Chem.* **2024**, *22*, doi:10.2174/0118715257251651231212045407.
